# Preliminary Formulation-Dependent Angiogenesis-Related and Early Osteogenic Responses to Three-Dimensional Bioprinted Hydroxyapatite–Acrylated Palm Olein Scaffolds: An In Vitro Study

**DOI:** 10.3390/ijms27177531

**Published:** 2026-08-22

**Authors:** Xi Chen, Nik Madihah Nik Azis, Syafira Masri, Masfueh Razali

**Affiliations:** 1 Dean’s Office, Faculty of Dentistry, Universiti Kebangsaan Malaysia, Jalan Raja Muda Abdul Aziz, Kuala Lumpur 50300, Malaysia; p138218@siswa.ukm.edu.my; 2 Department of Restorative Dentistry, Faculty of Dentistry, Universiti Kebangsaan Malaysia, Jalan Raja Muda Abdul Aziz, Kuala Lumpur 50300, Malaysia; 3 Department of Medical Informatics, RCSI & UCD Malaysia Campus (RUMC), Tingkat 2B, Tingkat 8 dan Tingkat 11B, No. 54, Jalan Sultan Ahmad Shah, George Town 10050, Malaysia; mm.nsyafira@rumc.edu.my

**Keywords:** 3D bioprinting, hydroxyapatite, acrylated palm olein, angiogenesis, osteogenesis, periodontal regeneration

## Abstract

Periodontal and alveolar bone regeneration requires coordinated angiogenic and osteogenic responses supported by biomimetic scaffolds. This study compared three-dimensional bioprinted hydroxyapatite–acrylated palm olein (3D-HA–APO) scaffold formulations containing 5%, 7% and 10% (*w*/*v*) hydroxyapatite (HA), designated F1, F2 and F3, respectively. Human umbilical vein endothelial cells were cultured on the scaffolds, and background-corrected soluble vascular endothelial growth factor (VEGF) concentrations in culture supernatants were quantified by enzyme-linked immunosorbent assay (ELISA). Angiogenic-related responses of human periodontal ligament stem cells were assessed by VEGF and cluster of differentiation 31 (CD31) immunofluorescence after endothelial induction, while alkaline phosphatase (ALP) activity in construct lysates was used as an indicator of early osteogenic activity. Soluble VEGF concentrations increased from F1 to F3, with significant differences between all formulations. VEGF-associated signal proportions differed among formulations, with F3 significantly higher than F1. CD31-associated signal proportions increased progressively from F1 to F3, with significant differences between all formulation pairs. ALP activity increased over time in all scaffold groups, and F3 generally showed the highest activity from Day 4 onwards. Overall, F3 showed the most favourable formulation-level profile across the selected angiogenic-related and early osteogenic outcomes.

## 1. Introduction

Periodontitis causes progressive destruction of the gingiva, periodontal ligament, cementum and alveolar bone, and remains a major cause of tooth loss in adults [1]. Although conventional periodontal therapy can control inflammation and limit further tissue breakdown, predictable reconstruction of the entire periodontal attachment apparatus remains difficult. Functional regeneration requires coordinated formation of alveolar bone, cementum and periodontal ligament fibres rather than bone fill alone [2,3,4]. This challenge is particularly evident in irregular or extensive periodontal defects, where conventional particulate grafts may provide inadequate structural stability and limited conformity to the defect geometry.

Three-dimensional (3D) bioprinting offers a potential means of producing defect-specific scaffolds with controlled architecture, interconnected pores and reproducible material distribution. Hydroxyapatite (HA) and tricalcium phosphate-based scaffolds have shown potential for craniofacial and intraoral bone regeneration because their mineral composition resembles that of native bone and supports osteoconduction [5]. Nevertheless, ceramic-rich constructs may have limited printability and handling properties, while inadequate vascularisation within larger scaffolds can restrict cell survival and subsequent tissue formation [6]. Composite systems that combine a mineral phase with a printable polymeric matrix may therefore provide a more suitable balance between fabrication performance and biological activity.

Acrylated palm olein (APO) is a palm oil-derived polymeric material that has been investigated in several biomedical and additive-manufacturing applications [7]. Its reactive functional groups permit crosslinking, while its polymeric properties can support scaffold formation and structural stability when combined with inorganic components [8,9,10]. APO also represents a renewable alternative to several conventional synthetic polymers. Previous work has primarily examined its polymerisation, printability and general cytocompatibility. However, its biological contribution within HA-containing 3D-printed scaffolds remains less well characterised, particularly in relation to cellular responses relevant to periodontal and alveolar bone regeneration.

The regenerative performance of a bone scaffold depends on more than osteogenic activity alone. Angiogenesis is required to provide oxygen, nutrients and regulatory signals to cells within the developing tissue. Vascular endothelial growth factor (VEGF) is a major regulator of endothelial cell migration, proliferation and vascular development, whereas platelet endothelial cell adhesion molecule-1 (PECAM-1/CD31) contributes to endothelial cell adhesion and vessel organisation. Angiogenic and osteogenic processes are closely linked during bone repair, as endothelial cells influence osteogenic differentiation and developing bone tissue, in turn, provides signals that support vascular growth [11,12]. Evaluation of VEGF and CD31-associated responses together with alkaline phosphatase (ALP) activity can therefore provide complementary information on selected early biological responses to a regenerative scaffold [13].

Variation in HA content alters the overall formulation of composite scaffolds and may affect both their physicochemical properties and cellular responses. Changes in mineral loading can alter rheological behaviour, printability and the resulting scaffold architecture and surface characteristics [14,15,16]. Such formulation-related changes may, in turn, modify the physical microenvironment experienced by attached cells. Consequently, biological differences among formulations containing different HA concentrations cannot necessarily be attributed to HA content alone. However, limited evidence is available on the angiogenic-related and early osteogenic responses associated with APO-based scaffold formulations containing different HA levels. Previous work from our research group reported preliminary structural and biological evaluation of an oil-based HA bioink platform, together with exploratory assessment of its angiogenic potential in vivo [17,18]. Drawing on this previously developed platform, three candidate formulations containing 5%, 7% and 10% (*w*/*v*) HA were selected for comparative biological evaluation in the present study.

Human umbilical vein endothelial cells (HUVECs) were used to evaluate scaffold-associated soluble VEGF levels, while VEGF- and CD31-associated signals were assessed in hPDLSCs following endothelial induction. Early osteogenic activity was evaluated using an ALP assay. Although the formulations were derived from a previously developed material platform, formulation-matched quantitative physicochemical characterisation was not included in the present study. The observed differences were therefore interpreted at the formulation level rather than as isolated effects of HA concentration.

This study therefore compared angiogenic-related and early osteogenic responses among three 3D-HA–APO scaffold formulations containing 5%, 7% and 10% (*w*/*v*) HA. We hypothesised that the formulations would produce different VEGF-, CD31- and ALP-associated responses. The findings were intended to identify the formulation with the most favourable early biological response profile among those tested and to provide preliminary evidence for subsequent physicochemical, mechanistic and preclinical evaluation.

## 2. Results

### 2.1. Gross Appearance of the 3D-HA–APO Scaffolds

**Figure 1:**
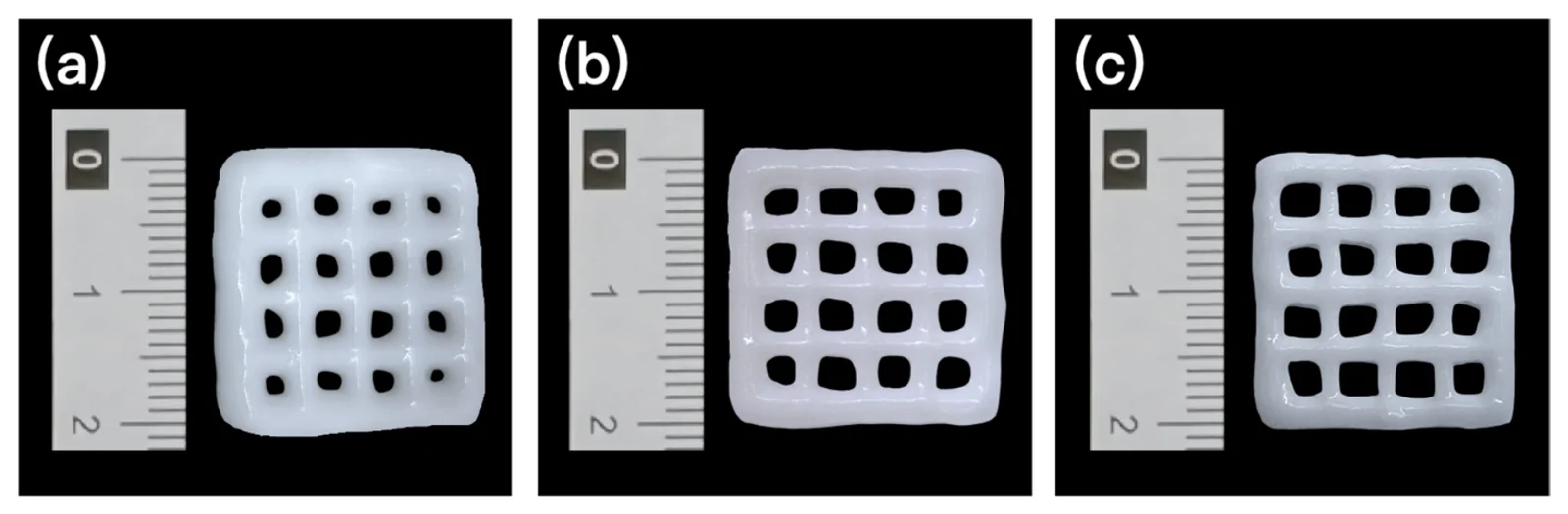
Representative gross appearance of three-dimensional hydroxyapatite–acrylated palm olein (3D-HA–APO) scaffolds printed using a predefined grid geometry. The scaffolds contained (**a**) 5% (*w*/*v*), (**b**) 7% (*w*/*v*) and (**c**) 10% (*w*/*v*) hydroxyapatite (HA).

Representative 3D-HA–APO scaffolds containing 5%, 7% and 10% (*w*/*v*) HA were fabricated using the same predefined grid geometry. By gross visual inspection, all three formulations formed square constructs with continuous filament deposition and regularly spaced pore-like openings (Figure 1). No obvious filament discontinuity or macroscopic structural collapse was observed immediately after printing and crosslinking.

### 2.2. Background-Corrected VEGF Concentrations in HUVEC Culture Supernatants

Background-corrected VEGF concentrations were determined in culture supernatants collected after 4 days. For each scaffold-containing group, the mean VEGF concentration of the corresponding scaffold-only controls was subtracted from each HUVEC-seeded scaffold value. The processed values for F1, F2 and F3 therefore represent soluble VEGF concentrations detected in HUVEC–scaffold culture supernatants after correction for medium-derived and scaffold-associated background.

A scaffold-free HUVEC-only condition was included as a two-dimensional (2D) reference. For this group, the mean VEGF concentration of the medium-only controls was subtracted from each HUVEC cell-only value. The resulting values were reported as the HUVEC-only reference and were not included in the inferential comparison among the scaffold formulations.

**Figure 2:**
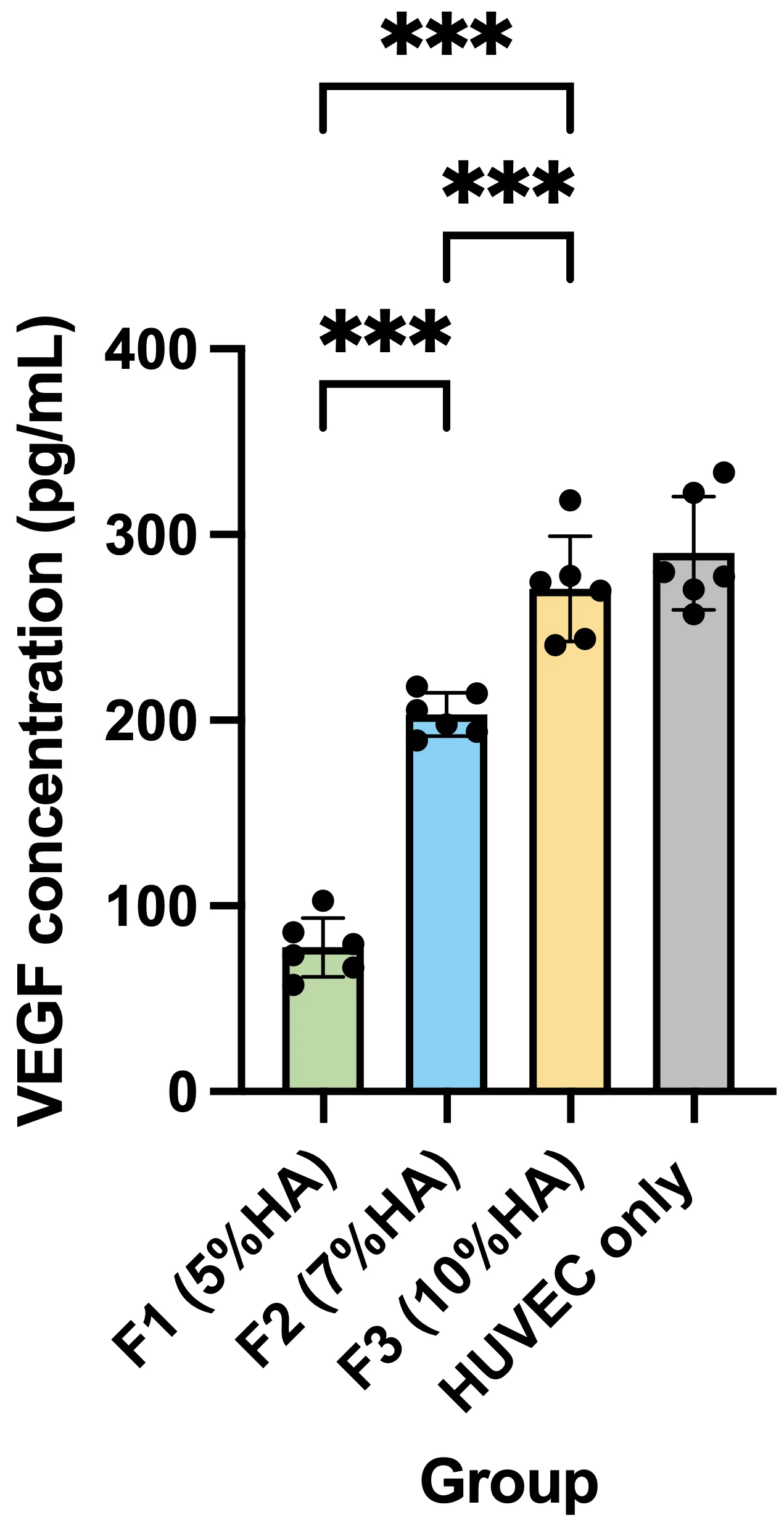
Background-corrected soluble vascular endothelial growth factor (VEGF) concentrations detected after 4 days of culture. For F1, F2 and F3, the mean VEGF concentration of the corresponding scaffold-only controls was subtracted from each human umbilical vein endothelial cell (HUVEC)-seeded scaffold value. The HUVEC-only reference was calculated by subtracting the mean VEGF concentration of the medium-only controls from each HUVEC cell-only value. F1, F2 and F3 contained 5%, 7% and 10% (*w*/*v*) hydroxyapatite (HA), respectively. Data are presented as mean ± standard deviation (SD) from six independently cultured biological samples (*N* = 6), with individual points representing the analysed biological replicate values. Statistical comparisons were performed among F1, F2 and F3 using one-way analysis of variance (ANOVA), *F*(2, 15) = 146.094, *p* < 0.001, η^2^ = 0.951, followed by Tukey’s honestly significant difference (HSD) test. The adjusted *p* values were <0.001 for F2 versus F1, F3 versus F1 and F3 versus F2. The HUVEC-only group was included as a descriptive scaffold-free reference and was not included in the inferential analysis. *** *p* < 0.001.

Background-corrected VEGF concentrations increased progressively from F1 to F3 (Figure 2). The mean concentration was 77.68 ± 15.83 pg/mL in F1, 203.16 ± 11.62 pg/mL in F2 and 270.86 ± 28.25 pg/mL in F3. The mean background-corrected concentration in the scaffold-free HUVEC-only reference was 290.08 ± 30.57 pg/mL. One-way analysis of variance (ANOVA) showed a significant overall difference among F1, F2 and F3, *F*(2, 15) = 146.094, *p* < 0.001, η^2^ = 0.951.

Tukey’s honestly significant difference (HSD) test showed significant differences between all scaffold formulations. VEGF concentration was significantly higher in F2 than in F1, with a mean difference of 125.48 pg/mL (*p* < 0.001). F3 was also significantly higher than F1 and F2, with mean differences of 193.18 pg/mL and 67.70 pg/mL, respectively (*p* < 0.001 for both comparisons). These findings demonstrate significant differences in background-corrected soluble VEGF concentrations among the tested formulations, with values increasing from F1 to F3 under the present HUVEC culture conditions. The representative standard curve, background control data, descriptive statistics, assumption testing and pairwise comparisons are provided in Figures S1–S3 and Tables S1–S3.

### 2.3. Endothelial-Associated Responses of hPDLSCs on 3D-HA–APO Scaffolds

**Figure 3:**
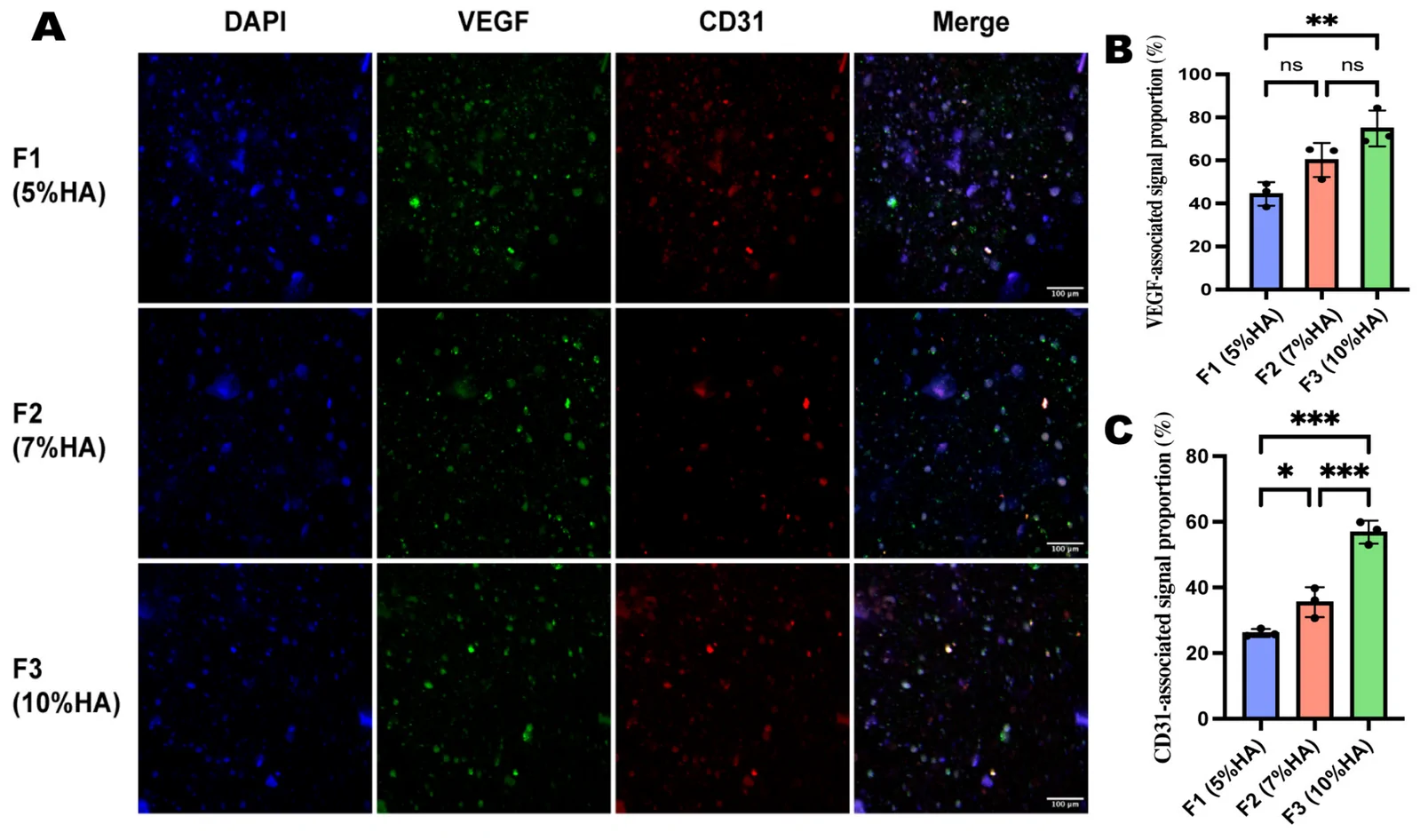
Endothelial-associated immunofluorescence signals in human periodontal ligament stem cells (hPDLSCs) cultured on three-dimensional hydroxyapatite–acrylated palm olein (3D-HA–APO) scaffolds after 21 days of endothelial induction. (**A**) Representative confocal maximum-intensity projection images of hPDLSCs cultured on F1, F2 and F3 scaffolds. F1, F2 and F3 contained 5%, 7% and 10% (*w*/*v*) hydroxyapatite (HA), respectively. Cells were stained for 4′,6-diamidino-2-phenylindole (DAPI), vascular endothelial growth factor (VEGF) and cluster of differentiation 31 (CD31). Scale bar = 100 µm. (**B**) VEGF-associated signal proportions relative to DAPI-detectable nuclear signals. (**C**) CD31-associated signal proportions relative to DAPI-detectable nuclear signals. Data are presented as mean ± standard deviation (SD) based on scaffold-level biological replicate values (*N* = 3 per formulation). Tukey’s HSD *p* values for VEGF-associated signals were 0.086 for F2 versus F1, 0.005 for F3 versus F1 and 0.110 for F3 versus F2. Corresponding *p* values for CD31-associated signals were 0.034 for F2 versus F1 and <0.001 for both F3 versus F1 and F3 versus F2. ns, not significant; * *p* < 0.05; ** *p* < 0.01; *** *p* < 0.001.

Representative immunofluorescence images and quantitative analyses of VEGF- and CD31-associated signals are shown in Figure 3.

After 21 days of endothelial induction, hPDLSCs cultured on F1, F2 and F3 scaffolds showed detectable VEGF- and CD31-associated immunofluorescence signals (Figure 3A). DAPI staining confirmed the presence of cells within the analysed scaffold regions. VEGF-associated signals appeared mainly as green cell-associated fluorescence, whereas CD31-associated signals appeared as red clustered fluorescence within the 3D scaffold environment. The merged images showed spatial association of VEGF- and CD31-associated signals with DAPI-detectable nuclei.

Qualitative control imaging supported the interpretation of the staining results. HUVEC positive controls showed clear VEGF and CD31 staining, whereas secondary antibody-only controls showed minimal background fluorescence. Non-induced hPDLSC controls showed weaker VEGF- and CD31-associated signals than the induced scaffold groups. Representative control images and additional induced scaffold images are provided in Figures S4–S7.

Quantitative analysis was performed only for the endothelial-induced F1–F3 scaffold groups. The VEGF- and CD31-associated signal proportions were calculated relative to the corresponding DAPI-detectable nuclear signal count in each image and then averaged to generate scaffold-level biological replicate values.

VEGF-associated signal proportions increased from F1 to F3 (Figure 3B). One-way ANOVA showed a significant overall difference among formulations, *F*(2, 6) = 12.885, *p* = 0.007, η^2^ = 0.811. Tukey’s HSD test showed that F3 was significantly higher than F1 (*p* = 0.005), whereas F2 versus F1 (*p* = 0.086) and F3 versus F2 (*p* = 0.110) were not significant.

CD31-associated signal proportions showed a clearer graded difference among the formulations (Figure 3C). One-way ANOVA showed a significant difference among formulations, *F*(2, 6) = 64.541, *p* < 0.001, η^2^ = 0.956. Tukey’s HSD test showed significant differences between all formulation pairs: F2 versus F1, *p* = 0.034; F3 versus F1, *p* < 0.001; and F3 versus F2, *p* < 0.001.

Together, these results demonstrate differences in VEGF- and CD31-associated signal proportions among the tested formulations. F3 showed the strongest endothelial-associated signal profile, with the clearest between-formulation differences observed for CD31. Additional representative immunofluorescence images and control images are provided in Figures S4–S7, with detailed statistical results provided in Tables S4–S8.

### 2.4. ALP Activity During Osteogenic Induction

**Figure 4:**
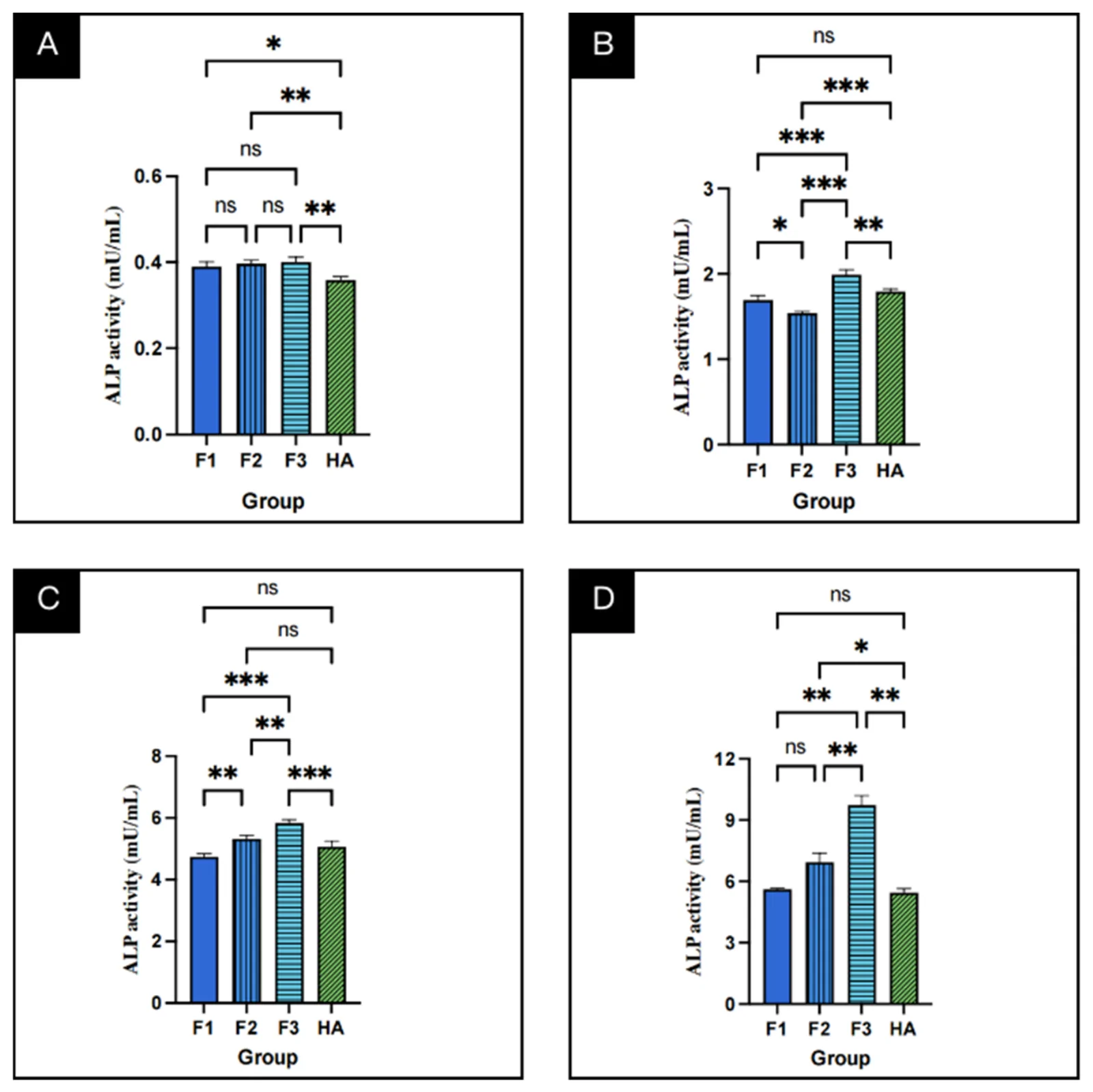
Time-point-specific comparisons of alkaline phosphatase (ALP) activity measured in clarified lysates from human periodontal ligament stem cells (hPDLSCs) cultured on three-dimensional hydroxyapatite–acrylated palm olein (3D-HA–APO) scaffolds and the 100% hydroxyapatite (HA) reference construct. ALP activity was compared among F1, F2, F3 and HA at (**A**) Day 1, (**B**) Day 4, (**C**) Day 7 and (**D**) Day 10 of osteogenic induction. F1, F2 and F3 contained 5%, 7% and 10% (*w*/*v*) HA, respectively. Data are presented as mean ± standard deviation (SD) based on biological replicate values (*N* = 3 per group at each time point). Day 1, Day 4 and Day 7 were analysed using one-way analysis of variance followed by Tukey’s HSD test, whereas Day 10 was analysed using Welch ANOVA followed by Games–Howell comparisons. Significant pairwise *p* values were as follows: Day 1, HA versus F1, *p* = 0.015; HA versus F2, *p* = 0.004; HA versus F3, *p* = 0.003. Day 4, F2 versus F1, *p* = 0.010; F3 versus F1, *p* < 0.001; F3 versus F2, *p* < 0.001; HA versus F2, *p* < 0.001; HA versus F3, *p* = 0.002. Day 7, F2 versus F1, *p* = 0.003; F3 versus F1, *p* < 0.001; F3 versus F2, *p* = 0.006; HA versus F3, *p* < 0.001. Day 10, F3 versus F1, *p* = 0.009; F3 versus F2, *p* = 0.006; HA versus F2, *p* = 0.048; HA versus F3, *p* = 0.003. Comparisons not listed were not statistically significant. ns, not significant; * *p* < 0.05; ** *p* < 0.01; *** *p* < 0.001.

ALP activity in construct lysates was assessed as an indicator of early osteogenic responses in hPDLSCs cultured on induced F1, F2 and F3 scaffolds and the 100% HA reference construct over 10 days (Figure 4). ALP activity increased over time in all groups, with the lowest values observed on Day 1 and higher values detected at later induction time points.

Significant differences in ALP activity were detected among the four groups at all evaluated time points. On Day 1, F1, F2 and F3 showed comparable ALP activity, whereas all three 3D-HA–APO scaffold groups showed higher ALP activity than the HA reference construct (Figure 4A). By Day 4, between-group differences became more evident, with F3 showing higher ALP activity than F1, F2 and HA (Figure 4B). On Day 7, F3 remained higher than F1, F2 and HA, and F2 was also higher than F1 (Figure 4C). On Day 10, F3 showed the highest ALP activity among all groups, while F2 was also higher than HA (Figure 4D).

Two-way ANOVA showed significant effects of day, *F*(3, 32) = 3331.181, *p* < 0.001, partial η^2^ = 0.997, and scaffold group, *F*(3, 32) = 147.679, *p* < 0.001, partial η^2^ = 0.933. A significant Day × Scaffold Group interaction was also detected, *F*(9, 32) = 77.505, *p* < 0.001, partial η^2^ = 0.956, indicating that differences in ALP activity among the groups varied over time. Representative ALP assay standard curves are provided in Figure S8. Detailed descriptive statistics, assumption testing, two-way ANOVA results, time-point-specific omnibus tests and post hoc comparisons are provided in Tables S9–S16. Supportive control data, including induced and non-induced scaffold comparisons and quantifiable 2D-induced ALP activity, are provided in Figure S9 and Tables S17 and S18.

**Figure 5:**
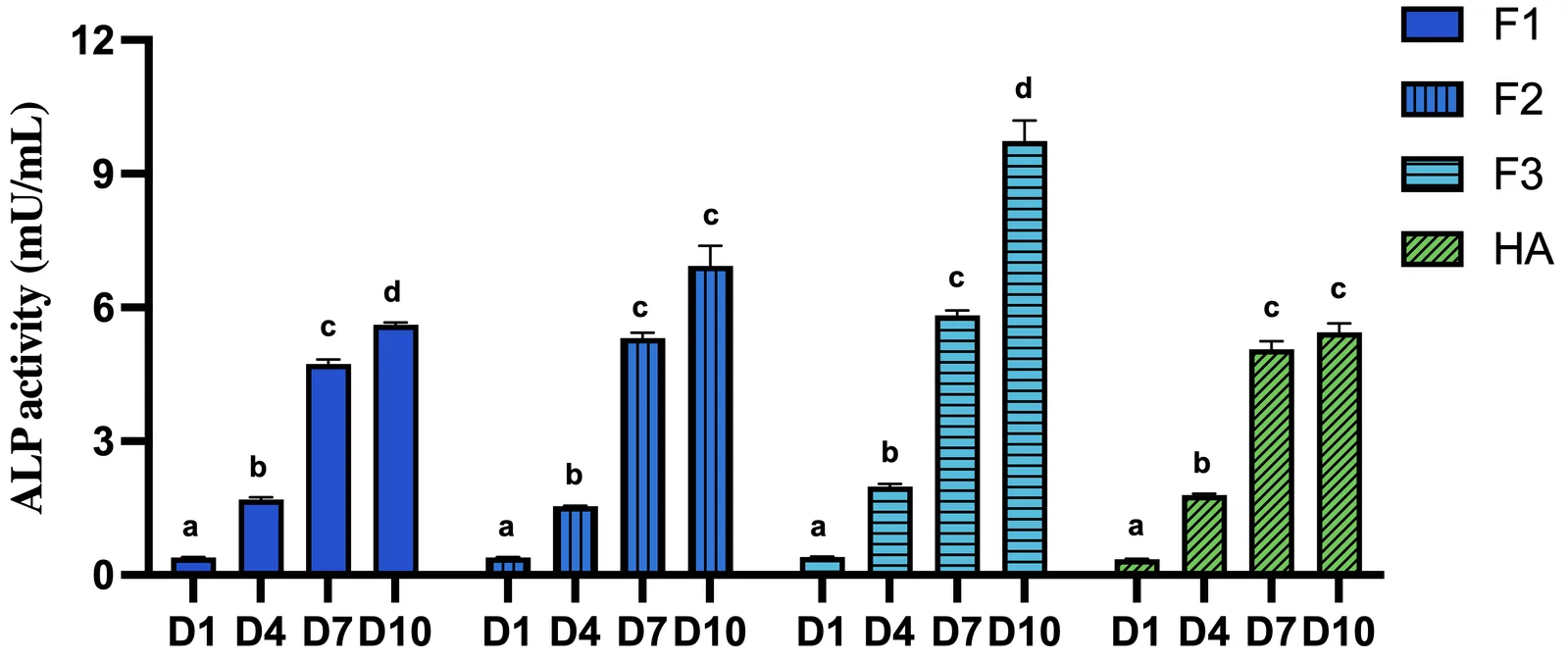
Temporal progression of alkaline phosphatase (ALP) activity measured in clarified lysates from human periodontal ligament stem cell (hPDLSC)-seeded three-dimensional hydroxyapatite–acrylated palm olein (3D-HA–APO) scaffolds and the 100% hydroxyapatite (HA) reference construct. ALP activity was measured on Days 1, 4, 7 and 10 of osteogenic induction. F1, F2 and F3 contained 5%, 7% and 10% (*w*/*v*) HA, respectively. Data are presented as mean ± standard deviation (SD) based on biological replicate values (*N* = 3 per group at each time point). Temporal differences within F1 were analysed using one-way analysis of variance followed by Tukey’s HSD test. Temporal differences within F2, F3 and the HA reference group were analysed using Welch ANOVA followed by Games–Howell comparisons. Different lowercase letters indicate statistically significant differences among time points within the same scaffold group. Time points sharing the same letter were not significantly different. Letters should be interpreted within each scaffold group only and not as comparisons between different groups. Exact within-group pairwise *p* values are provided in Table S19.

The temporal profile of ALP activity is shown in Figure 5. ALP activity increased significantly from Day 1 to the later induction time points in all scaffold groups. F1 and F3 showed significant increases between each consecutive time point, whereas the differences between Day 7 and Day 10 were not significant for F2 (*p* = 0.051) or the HA reference construct (*p* = 0.217). Among the 3D-HA–APO formulations, F3 showed the largest overall increase and the highest ALP activity at Day 10.

Overall, ALP activity differed among the scaffold formulations under matched initial seeding and osteogenic induction conditions. F3, the formulation containing 10% (*w*/*v*) HA, showed the highest ALP activity from Day 4 onwards.

## 3. Discussion

### 3.1. Principal Findings

The present study demonstrated formulation-dependent differences in selected angiogenic-related and early osteogenic responses among 3D hydroxyapatite–acrylated palm olein (3D-HA–APO) scaffolds. Background-corrected soluble vascular endothelial growth factor (VEGF) levels in human umbilical vein endothelial cell (HUVEC) culture supernatants increased from F1 to F3. In endothelial-induced human periodontal ligament stem cells (hPDLSCs), VEGF- and CD31-associated signal proportions increased across the formulations from F1 to F3, with clearer between-formulation differences observed for CD31. Alkaline phosphatase (ALP) activity increased over time in all scaffold groups, with F3 generally showing the highest activity from Day 4 onwards.

Taken together, the 10% (*w*/*v*) HA formulation showed the most favourable selected early biological response profile among the tested scaffold formulations. Although F3 showed the strongest overall response profile, the observed differences are unlikely to reflect HA concentration alone. Variation in mineral loading can influence ink rheology, printability and the resulting scaffold geometry [14,15,16], while associated changes in surface characteristics and mechanical behaviour may also affect cell–material interactions [19,20]. Because these properties were not quantified across the three formulations, the results are best interpreted as formulation-level differences rather than a specific HA dose–response relationship.

### 3.2. Soluble VEGF Concentrations in HUVEC Culture Supernatants

#### 3.2.1. Formulation-Dependent VEGF Response

Background-corrected soluble VEGF levels increased progressively from F1 to F3, indicating a graded formulation-dependent response rather than random variation among the scaffold groups. However, this trend should be interpreted at the formulation level and does not establish an isolated effect of HA concentration.

Endothelial responses to HA-containing scaffolds are unlikely to depend on nominal HA concentration alone. Particle size, surface chemistry, incorporation within the matrix and the extent of cell–material contact can all influence biological responses. Previous studies have reported that some forms of internalisable HA nanoparticles may suppress angiogenesis-related signalling, whereas endothelial responses can vary according to HA particle size, concentration and scaffold presentation [21,22]. These apparently contrasting findings indicate that HA does not produce a uniformly positive or linear angiogenic effect. Instead, its influence depends on how the mineral phase is presented within the scaffold.

In the present study, HA was incorporated into an APO-containing composite rather than delivered as free particles. Differences among F1, F2 and F3 may therefore have involved variation in surface characteristics, mineral availability, scaffold geometry and cell–material interactions. HA-containing scaffold systems have previously been shown to influence endothelial organisation and vascularisation-related outcomes, supporting the view that the mineral phase can contribute to the local biological environment [23]. Nevertheless, soluble VEGF measurements alone cannot establish whether the higher values in F3 resulted primarily from increased cellular secretion, altered cellular uptake, protein retention by the scaffold or a combination of these processes.

Functional assays would be required to determine whether the observed VEGF pattern translates into improved angiogenic performance. Endothelial migration, tube formation and network-formation assays would provide more direct evidence of vascular function. Future studies combining physicochemical characterisation with biological evaluation would help clarify which scaffold properties contributed most to the observed formulation-dependent responses [24].

#### 3.2.2. Interpretation of Background-Corrected VEGF Measurements

A major consideration in interpreting the VEGF results was the presence of exogenous growth factors in endothelial growth medium. Endothelial-specific media commonly contain VEGF or related supplements to maintain HUVEC growth and function, and VEGF-containing endothelial culture systems have been used to support HUVEC maintenance and angiogenic-related responses [25,26]. Consequently, VEGF detected in the culture supernatant may represent both medium-derived and cell-associated contributions.

To reduce this source of confounding, formulation-specific scaffold-only controls were processed in parallel and subtracted from the corresponding HUVEC–scaffold measurements. The medium-only control was similarly subtracted from the scaffold-free HUVEC reference. This approach was more appropriate than comparing raw supernatant values because HA-containing materials can adsorb or retain proteins through surface interactions involving calcium- and phosphate-rich binding sites [27,28].

Previous work has shown that growth factors can remain associated with HA surfaces and retain biological activity [27,28]. Scaffold microstructure and physicochemical properties, including porosity, surface characteristics and swelling behaviour, may also influence the diffusion and retention of soluble proteins [24]. Therefore, the measured supernatant VEGF concentrations may have reflected both HUVEC-associated VEGF production and interactions between VEGF and the scaffold matrix.

Scaffold-specific blank correction reduced the contribution of cell-free material effects and provided a more meaningful basis for comparing soluble VEGF among formulations. However, it did not fully resolve the dynamic behaviour of VEGF within the culture system. The measured supernatant concentration may still have been influenced by cellular secretion, uptake, adsorption, retention and re-release. Future studies assessing VEGF adsorption, release and scaffold-bound VEGF would help distinguish these mechanisms.

### 3.3. Endothelial-Associated Responses of hPDLSCs

#### 3.3.1. Interpretation of VEGF- and CD31-Associated Signals

VEGF- and CD31-associated signals provided complementary information on the endothelial-associated response of hPDLSCs cultured on 3D-HA–APO scaffolds. VEGF-associated signal proportions increased from F1 to F3, although only the comparison between F3 and F1 reached statistical significance. This indicates an overall graded pattern across the formulations, but the difference was most clearly detectable between F1 and F3.

VEGF is not specific to endothelial cells. Periodontal ligament stem cells and other dental-derived mesenchymal cells can express or secrete VEGF in response to regenerative or environmental stimuli [29,30]. The presence of weak VEGF-associated signals in non-induced hPDLSCs was therefore biologically plausible. In this study, VEGF should be interpreted as an indicator of angiogenic-related activity rather than direct evidence of endothelial lineage commitment.

CD31 provided a more endothelial-associated readout because it is a junctional adhesion molecule commonly enriched at endothelial cell membranes and cell–cell contacts [31]. CD31-associated signal proportions showed significant differences between all scaffold formulation pairs and increased progressively from F1 to F3. The stronger and more consistent CD31 pattern indicates that F3 showed the most pronounced endothelial-associated signal profile among the tested scaffolds.

The relatively discontinuous appearance of CD31 staining does not necessarily indicate unsuccessful endothelial induction. Formation of continuous endothelial junctions depends on cell maturity, cell–cell contact, culture duration and scaffold architecture. After 21 days, hPDLSCs may have acquired endothelial-associated features without developing a fully mature monolayer-like phenotype. Previous stem-cell differentiation studies have similarly used CD31 as an indicator of endothelial acquisition, although its expression alone does not confirm complete functional endothelial differentiation [32].

#### 3.3.2. Formulation-Related Influences on Endothelial-Associated Responses

One possible explanation for higher VEGF- and CD31-associated signals is that the scaffold formulations differed in cell retention or distribution rather than marker response. Cells within 3D constructs may attach unevenly, migrate into pores or be located at different depths, leading to variation in the number of detectable nuclei within each region of interest (ROI) [33].

The present data do not support a simple cell-number-driven explanation. F1 showed the highest mean number of DAPI-detectable nuclear signals but the lowest VEGF- and CD31-associated signal proportions. In contrast, F2 and F3 showed lower and broadly comparable nuclear counts while exhibiting higher marker-associated proportions. This pattern suggests that the observed formulation differences were not merely a consequence of more detectable cells in F3.

Normalising marker-associated particles to DAPI-detectable nuclear signals therefore allowed the results to be expressed relative to the detectable cell population within each ROI. This reduced the influence of variation in cell number, spatial distribution and imaging depth. Although such normalisation cannot remove all optical or biological variability, it provides a more informative comparison than absolute particle counts alone.

The stronger signal profile observed in F3 indicates that the three scaffold formulations created distinct microenvironments for endothelial-associated responses. Variation in mineral loading can influence surface characteristics, cell adhesion, local ion availability and matrix-mediated signalling [23,34], and these interconnected changes may collectively contribute to the graded response observed from F1 to F3. Within the tested formulation range, F3 was associated with the most favourable microenvironment for the selected endothelial-associated markers.

#### 3.3.3. Methodological Considerations for 3D Immunofluorescence Quantification

HUVECs were used as a positive staining control to confirm antibody performance and expected signal localisation. They showed cytoplasmic VEGF-associated staining and membrane-associated CD31 staining under 2D culture conditions, consistent with the known intracellular roles of VEGF and the membrane/junctional localisation of CD31 [31,35]. This control was not used as a quantitative comparator because direct comparison between a 2D monolayer and cells embedded in a mineral-containing 3D scaffold would be affected by substantial structural and optical differences.

Within 3D-HA–APO scaffolds, VEGF- and CD31-associated signals often appeared punctate or clustered rather than forming the continuous patterns commonly observed in endothelial monolayers. This morphology is plausible because cells within 3D matrices adopt non-planar orientations and form spatially heterogeneous cell–cell and cell–matrix contacts [36,37]. HA-containing constructs also introduce refractive-index heterogeneity and light scattering, which can attenuate or fragment fluorescent signals across depth [38].

For this reason, fluorescence intensity alone may be difficult to compare reliably in mineral-rich 3D scaffolds. The present study used a standardised particle-based workflow under fixed acquisition and analysis settings [39]. Marker-associated particles were quantified using consistent thresholds and size filters and were normalised to DAPI-detectable nuclear signals within the same ROI. This rule-based approach reduced reliance on subjective manual counting and supported internal comparison among scaffold formulations.

### 3.4. Osteogenic Responses Under Induction

#### 3.4.1. Formulation-Dependent Differences in ALP Activity

ALP activity showed both formulation- and time-dependent differences during osteogenic induction. Among the 3D-HA–APO formulations, F3 generally produced the strongest response, particularly from Day 4 onwards. At Day 1, ALP activity was comparable among F1, F2 and F3. Clearer formulation-dependent differences emerged at later time points, when F3 showed significantly higher activity than F1 and F2.

The observed pattern is consistent with previous evidence that HA-containing substrates can support osteogenic differentiation. Through mineral chemistry, calcium and phosphate availability, surface reactivity and micro- or nanotopographical cues, HA may promote early osteogenic responses, including enhanced ALP activity and osteogenic marker expression, in mesenchymal cell populations such as periodontal ligament-derived stem cells [34,40].

The 100% HA reference construct did not consistently produce the highest ALP activity despite its greater mineral content. Unlike F1–F3, it lacked the APO-containing matrix and printable scaffold architecture and therefore served as a clinically relevant HA-based reference rather than a formulation-matched comparator.

The present study was not designed to establish an optimal HA concentration or a concentration–response relationship. Instead, it compared three candidate scaffold formulations selected during material development to identify the formulation showing the most favourable overall biological performance under the present experimental conditions. In extrusion-based scaffold development, formulation selection requires balancing biological objectives with processability, as HA loading can influence ink rheology, extrusion behaviour, printability and the resulting scaffold features [14,15]. Within this predefined formulation set, F3 consistently showed the strongest early osteogenic response. The finding that the 100% HA reference construct did not consistently produce the highest ALP activity indicates that biological performance depended on the overall scaffold formulation rather than mineral content alone. Accordingly, the present findings should not be interpreted as evidence that higher HA concentrations would necessarily produce greater biological responses or that 10% (*w*/*v*) HA represents an optimal HA concentration. Rather, F3 should be regarded as the best-performing formulation among the three candidate formulations evaluated in this study.

#### 3.4.2. Time-Dependent Progression of ALP Activity

ALP activity increased over time in all scaffold groups, confirming that osteogenic-related enzyme activity developed during the induction period. The response was low at Day 1 and rose progressively at later time points, although the temporal pattern differed among formulations. F1 and F3 continued to increase significantly from Day 7 to Day 10, whereas F2 and the HA block showed a relative plateau during the later phase.

Among the printable 3D-HA–APO scaffolds, F3 showed the greatest increase in ALP activity and consistently produced the highest values from Day 4 onwards, indicating a more favourable early osteogenic response during the experimental period. However, increased ALP activity alone does not establish progression to matrix maturation or mineralisation. Whether the favourable early osteogenic response observed in F3 is maintained during later stages of differentiation remains to be determined using mid- and late-stage osteogenic markers, such as RUNX2, OCN and OPN, together with direct mineralisation assays including Alizarin Red S staining [41].

Differences in temporal progression are biologically plausible because ALP does not reach a fixed peak across all cell and scaffold systems; previous osteogenic studies have assessed ALP activity across different early induction windows depending on the cell type and scaffold model [42,43,44]. In some models, ALP activity continues to increase during early differentiation, whereas in others it plateaus or declines as cells progress towards matrix maturation and mineralisation. The persistent Day 10 response in F3 may therefore indicate continued osteogenic enzyme activation within the present experimental window rather than an earlier transition to a later maturation phase.

The 100% HA block did not consistently outperform the composite formulations. From Day 4 onwards, F3 showed higher ALP activity than the HA block, and F2 also showed a higher mean response at later time points. These findings indicate that the HA–APO composite formulations, particularly F3, supported higher construct-level ALP activity than the HA reference material under the present experimental conditions, consistent with a more favourable early osteogenic response profile. Although these findings do not constitute clinical evidence, they provide a useful basis for further material optimisation and preclinical evaluation.

### 3.5. Methodological and Translational Considerations

The 2D controls included in this study served as biological references and assay benchmarks rather than direct structural equivalents of the 3D scaffold environment. Cell behaviour in 2D and 3D systems differs in terms of spatial organisation, cell–matrix contact, nutrient diffusion, surface exposure and sample processing [36,37,45]. For this reason, the primary interpretation focused on comparisons among F1, F2 and F3 under matched 3D conditions.

This distinction was particularly important in the VEGF and ALP analyses. For the scaffold-containing groups, formulation-specific background controls were used to account for matrix-associated effects. In contrast, scaffold-free reference cultures were corrected using medium-only controls. The resulting values were therefore appropriate within their respective experimental contexts but were not treated as directly equivalent across 2D and 3D formats.

In the ALP assay, scaffold-derived lysates were clarified by high-speed centrifugation before analysis, consistent with previous ALP studies using cell-seeded hydrogel or three-dimensional scaffold samples [46]. Nevertheless, minor matrix-associated residues may have remained in the supernatants and contributed to assay background or other matrix-related effects. All scaffold groups were prepared, processed and analysed using the same lysate preparation, centrifugation and assay procedures to standardise sample handling across groups, although residual matrix interference could not be completely excluded. Future studies could further evaluate matrix interference, protein recovery efficiency and endpoint normalisation to total protein or DNA content.

### 3.6. Limitations and Future Directions

The present study has several limitations. First, biological evaluation was based on a focused set of angiogenic-related and osteogenic-related outcomes. VEGF, CD31 and ALP provided useful evidence of endothelial-associated signalling and early osteogenic activity, but they did not define the underlying molecular pathways, functional angiogenesis or later stages of osteogenic maturation and mineralisation. Future studies should therefore include broader gene and protein panels, pathway-related analyses, functional angiogenesis assays and additional evaluation of later osteogenic differentiation and mineralisation, including Alizarin Red S staining.

Second, the angiogenic-related and osteogenic-related responses were assessed in separate experimental systems. Although F3 showed the most favourable pattern across both biological domains, the study could not determine whether the two responses were mechanistically connected. Co-culture models involving endothelial cells and hPDLSCs, combined with longitudinal and spatial analyses, would provide a more direct means of examining angiogenesis–osteogenesis coupling within the same scaffold environment.

Third, the study used controlled in vitro mono-culture models and cell-seeded prefabricated scaffolds. These models enabled formulation-to-formulation comparison, but they did not reproduce the complex inflammatory microenvironment of periodontal disease, the interactions among multiple resident and immune cell populations, mechanical loading or vascular infiltration that occur during periodontal wound healing. Cell distribution within the scaffolds may also have been heterogeneous, and this model differs from a true cell-laden bioink in which cells are encapsulated throughout the printed construct. Future development could therefore explore cell-laden printing approaches, provided that printing pressure, crosslinking conditions and post-printing cell viability are carefully optimised.

Fourth, the VEGF and ALP measurements were not normalised to endpoint cell abundance, DNA content or total protein, and formal viability or proliferation assays were not performed. Consequently, the higher VEGF and ALP values observed in some scaffold formulations may reflect differences in endpoint cell abundance, biological activity, or a combination of both. Therefore, the present findings should be interpreted as construct-level biological responses rather than per-cell biological activity. Future studies should combine endpoint normalisation with direct cytocompatibility assessment to distinguish overall construct performance from cell-number-associated effects.

Fifth, formulation-matched quantitative physicochemical characterisation was not included in the present study. Although F1, F2 and F3 were fabricated using the same nominal printing design and process conditions, this does not establish equivalence in filament dimensions, pore architecture, surface morphology, stiffness, swelling, degradation or mineral release. Any differences in these properties may have independently influenced cell attachment, distribution and biological responses. Consequently, the observed differences cannot be attributed specifically to HA concentration and should be interpreted as associations with the complete scaffold formulations. Future studies should evaluate material and biological outcomes in the same scaffold batches to determine how physicochemical properties relate to the observed cellular responses.

## 4. Materials and Methods

### 4.1. Ethics Statement

This study was conducted within the scope of a broader research project approved by the Universiti Kebangsaan Malaysia Research Ethics Committee (approval no. UKM PPI/111/8/JEP-2022-774; approved on 13 January 2023). The present in vitro experiments used commercially sourced human umbilical vein endothelial cells and human periodontal ligament stem cells. No human participants were directly recruited, and no identifiable personal or clinical data were collected.

### 4.2. Study Design and Experimental Groups

This in vitro study comprised three experimental activities (namely, A1, A2 and A3), each evaluating a different aspect of the biological performance of 3D bioprinted hydroxyapatite–acrylated palm olein (3D-HA–APO) scaffolds. The scaffolds were formulated with three concentrations of hydroxyapatite (HA): 5%, 7% and 10% (*w*/*v*), designated F1, F2 and F3, respectively. Unless otherwise stated, HA refers to nano-sized hydroxyapatite throughout this study.

#### 4.2.1. Activity 1: VEGF Concentration Analysis by Enzyme-Linked Immunosorbent Assay (ELISA)

Activity 1 compared soluble vascular endothelial growth factor (VEGF) concentrations detected in human umbilical vein endothelial cell (HUVEC) culture supernatants after 4 days among the three 3D-HA–APO scaffold formulations. Commercially sourced pooled-donor HUVECs were obtained from Lonza (Walkersville, MD, USA). This time point was selected as an early assessment period commonly used for evaluating VEGF-related responses in endothelial cell culture [47].

The experimental groups were as follows:

1. 3D-HA–APO scaffold groups: HUVECs cultured with F1, F2 or F3 scaffolds containing 5%, 7% or 10% (*w*/*v*) HA, respectively.
2. Scaffold-only control groups: F1, F2 and F3 scaffolds incubated in endothelial growth medium without cells.
3. HUVEC cell-only group: HUVECs cultured in endothelial growth medium without a scaffold.
4. Medium-only group: Endothelial growth medium incubated without cells or scaffolds.

The scaffold-only and medium-only controls were included because the endothelial growth medium contained VEGF as part of its supplemented formulation, while calcium phosphate- and HA-containing materials may adsorb or retain VEGF and other soluble proteins [48]. The scaffold-only controls were therefore used to account for medium-derived and formulation-specific scaffold-associated background. The medium-only control was used to correct the scaffold-free HUVEC cell-only measurements. The resulting processed cell-only values were reported as the HUVEC-only reference.

The primary inferential comparison was performed among F1, F2 and F3. The HUVEC-only condition was included as a scaffold-free 2D reference and was not treated as a structurally equivalent comparator to the 3D scaffold groups.

#### 4.2.2. Activity 2: Endothelial-Associated Marker Assessment by Immunofluorescence

Activity 2 evaluated VEGF- and CD31-associated signals in commercially sourced human periodontal ligament stem cells (hPDLSCs; Celprogen, Torrance, CA, USA) after 21 days of endothelial induction on 3D-HA–APO scaffolds [49].

The experimental and control groups were as follows:

1. Endothelial-induced 3D-HA–APO groups: hPDLSCs cultured on F1, F2 and F3 scaffolds under endothelial induction conditions.
2. Two-dimensional HUVEC positive control: HUVECs cultured without scaffolds under standard endothelial culture conditions.
3. Non-induced hPDLSC controls: hPDLSCs maintained without endothelial induction under 2D and scaffold-based conditions.
4. Secondary antibody-only control: Endothelial-induced hPDLSC-seeded scaffolds processed without primary antibodies and stained only with the corresponding secondary antibodies and 4′,6-diamidino-2-phenylindole (DAPI).

The HUVEC positive control served as a mature endothelial reference and was used to confirm the expected detectability and localisation of VEGF- and CD31-associated fluorescence. The 2D and 3D non-induced hPDLSC controls were included to assess baseline marker-associated signals in the absence of endothelial induction. The secondary antibody-only control was used to evaluate non-specific secondary antibody binding and residual cell- or scaffold-associated background fluorescence under the same imaging conditions.

VEGF was assessed as an angiogenesis-related marker rather than as an endothelial-specific marker. CD31 was included as the more endothelial-associated marker, and the two readouts were interpreted together when evaluating the endothelial-associated response of hPDLSCs [35,50]. Quantitative comparisons were performed among the endothelial-induced F1, F2 and F3 groups, whereas the remaining controls were used primarily for qualitative validation.

#### 4.2.3. Activity 3: Osteogenic Differentiation Assessment

Activity 3 evaluated early osteogenic activity in hPDLSCs cultured on 3D-HA–APO scaffolds under osteogenic induction conditions. Samples were collected on Days 1, 4, 7 and 10, representing early time points commonly used to assess alkaline phosphatase (ALP)-related responses during in vitro osteogenic differentiation [51,52].

The experimental and control groups were as follows:

1. HA reference construct: A commercially manufactured 100% nano-hydroxyapatite block (GranuMaS™, Selangor, Malaysia).
2. Osteogenic-induced 3D-HA–APO groups: hPDLSCs cultured on F1, F2 and F3 scaffolds in osteogenic differentiation medium.
3. Non-induced 3D-HA–APO controls: hPDLSCs cultured on F1, F2 and F3 scaffolds in basal medium.
4. 2D controls: hPDLSCs cultured under induced and non-induced 2D conditions.

The induced groups were maintained in complete osteogenic differentiation medium prepared using the StemPro™ Osteogenesis Differentiation Kit (Gibco, Thermo Fisher Scientific, Grand Island, NY, USA) according to the manufacturer’s instructions. The non-induced groups were maintained in basal medium to distinguish induction-dependent effects from scaffold-associated background.

The 100% HA block was included as an HA-based reference construct because hydroxyapatite and related calcium phosphate materials are widely used in bone tissue engineering and clinical bone regeneration [53].

### 4.3. Replicate Structure and Experimental Units

In this study, *N* denotes the number of independent biological replicate values included in the statistical analysis, whereas *n* denotes the number of raw assay or image measurements obtained before averaging. Technical replicates and repeated image measurements from the same biological sample were not treated as independent experimental units. Where applicable, these measurements were averaged to generate one biological replicate value before inferential analysis. The replicate structures for Activities 1–3 are summarised in Figure S10.

#### 4.3.1. Replicate Structure for Activity 1

Each scaffold formulation comprised six independently cultured scaffold-associated biological samples (*N* = 6). Each culture supernatant was measured once by ELISA, resulting in six measurements per formulation (*n* = 6). No technical assay replicates were performed; therefore, each background-corrected VEGF measurement represented one biological replicate value used directly in the statistical analysis.

All collected supernatants were stored under the same conditions and analysed together in a single ELISA run to minimise inter-assay variation.

#### 4.3.2. Replicate Structure for Activity 2

Each scaffold formulation comprised three independently cultured biological samples (*N* = 3). Three regions of interest were analysed from each scaffold, resulting in nine image measurements per formulation (*n* = 9).

The three regions of interest were treated as repeated measurements within the same biological replicate rather than as independent samples. Their measurements were averaged to generate one biological replicate value for each scaffold before statistical analysis.

The HUVEC positive control, non-induced controls and secondary antibody-only controls were used for qualitative validation and were not included in the primary quantitative comparison among F1, F2 and F3.

#### 4.3.3. Replicate Structure for Activity 3

Each experimental group comprised three biological replicates at each time point (*N* = 3). Each lysate was measured in duplicate assay wells, resulting in six raw measurements per group at each time point (*n* = 6).

The duplicate measurements obtained from each lysate were averaged to generate one biological replicate value before inferential statistical analysis. The same replicate structure was applied to the induced scaffold groups, corresponding non-induced scaffold controls, the 100% HA reference construct and the 2D control groups.

### 4.4. Fabrication of 3D-HA–APO Scaffolds

#### 4.4.1. Preparation of the HA–APO Composite Ink

The HA–APO composite inks were prepared under aseptic conditions following the formulation approach described by Razali, Masri, Tajau and Mohd [17]. Nano-hydroxyapatite powder (GranuMaS™, Selangor, Malaysia), acrylated palm olein (Malaysian Nuclear Agency, Dengkil, Selangor, Malaysia), alginic acid sodium salt (Sigma-Aldrich, St. Louis, MO, USA), low-molecular-weight chitosan (Sigma-Aldrich, St. Louis, MO, USA), Tween 80 (Sigma-Aldrich, St. Louis, MO, USA), acetic acid (ScienCell Research Laboratories, Carlsbad, CA, USA) and sterile distilled water were used to prepare the composite inks. Three formulations containing 5%, 7% and 10% (*w*/*v*) nano-hydroxyapatite were designated F1, F2 and F3, respectively. The amounts of acrylated palm olein, alginate and chitosan were maintained constant across the three formulations.

Nano-hydroxyapatite was first dispersed in sterile distilled water under continuous magnetic stirring. Alginate was then added gradually and mixed until a homogeneous hydrogel base was obtained. Chitosan and acrylated palm olein were subsequently incorporated under continuous stirring. A 0.5% (*v*/*v*) acetic acid solution was added dropwise, with no more than 10 drops added per formulation, to facilitate chitosan dissolution and adjust the viscosity. All mixing steps were performed at room temperature. Mixing was continued until a smooth, cohesive and extrudable paste without visible phase separation or undissolved particles was obtained.

The prepared ink was transferred aseptically into a sterile 3 mL printing syringe using a Luer connector. Air entrapment was minimised by slow loading and gentle tapping. Printing was performed immediately after ink preparation to minimise phase separation and changes in viscosity.

#### 4.4.2. Extrusion-Based 3D Printing

The composite scaffolds were fabricated using a BIOX extrusion-based 3D bioprinter (CELLINK, Gothenburg, Sweden). Before printing, the printing chamber and platform were disinfected, and the internal ultraviolet light was operated for 15–30 min. The prepared inks were loaded into sterile syringes fitted with sterile extrusion nozzles with an inner diameter of 0.41 mm.

A grid-type architecture was selected from the preset design library of the BIOX system. The BIOX system generated the extrusion path and G-code according to the selected geometry and printing parameters. Printing was conducted at room temperature using a print speed of 15 mm/s and a pneumatic extrusion pressure of 20–30 kPa. The pressure was adjusted within this range to obtain continuous strands without excessive spreading or nozzle blockage.

Scaffold dimensions were selected according to the requirements of each experimental activity: 10 × 10 × 3 mm for Activity 1, 10 × 10 × 1 mm for Activity 2 and approximately 5 × 5 × 3 mm for Activity 3. The Activity 3 construct size was selected to provide a volume comparable to the 100% nano-hydroxyapatite reference block, which measured 6 mm in diameter and 3 mm in height.

All three formulations were printed using the same preset geometry and nominal printing parameters in an attempt to minimise manufacturing-related variation. The 100% nano-hydroxyapatite block was used in its original manufactured form and was not subjected to 3D printing. The principal steps of ink preparation, extrusion-based printing and construct formation are illustrated in Figure S11.

#### 4.4.3. Post-Printing Crosslinking, Washing and Storage

Immediately after printing, the scaffolds were crosslinked in situ using sterile 5% (*w*/*v*) calcium chloride solution prepared from calcium chloride dihydrate (Chemiz, Selangor, Malaysia). The solution was added dropwise until the printed constructs were fully immersed, and crosslinking was continued for 10–15 min. Calcium ions promoted ionic crosslinking of the alginate phase and stabilised the printed hydrogel structure, consistent with the calcium–alginate egg-box model [54].

The crosslinked scaffolds were washed repeatedly with sterile Dulbecco’s phosphate-buffered saline (DPBS; Elabscience, Houston, TX, USA) or sterile distilled water to remove residual calcium chloride and soluble components. The scaffolds were subsequently exposed to ultraviolet light for 20–30 min on each side for surface decontamination.

Scaffolds were stored in sterile sealed containers at 4 °C and used within approximately one week. Before cell seeding, they were equilibrated to the required culture temperature and pre-wetted with the appropriate culture medium, as described in the corresponding experimental sections.

### 4.5. Cell Culture

#### 4.5.1. HUVEC Culture

HUVECs were used at passages 3–5. Cells were maintained in complete Endothelial Growth Medium-2 (EGM-2), prepared by supplementing Endothelial Basal Medium-2 (EBM-2; Lonza, Walkersville, MD, USA) with the EGM-2 SingleQuots Supplement Pack (Lonza, Walkersville, MD, USA) according to the manufacturer’s instructions. Cultures were maintained at 37 °C in a humidified atmosphere containing 5% CO_2_, and the medium was replaced every 48 h.

For post-thaw recovery, cryovials were rapidly thawed in a 37 °C water bath until only a small ice crystal remained. The cell suspension was transferred into pre-warmed complete EGM-2 medium and seeded into T25 culture flasks. Following recovery, the cells were passaged once before being used in subsequent experiments. When cultures reached approximately 70–80% confluence, cells were washed with DPBS, detached using TrypLE™ Select Enzyme (1×; Gibco, Thermo Fisher Scientific, Waltham, MA, USA) and collected by centrifugation at 250× *g* for 5 min. The cell pellet was resuspended in complete EGM-2 medium and prepared for experimental seeding.

#### 4.5.2. hPDLSC Culture

hPDLSCs were used at passages 4–8. Cells were maintained in complete growth medium consisting of Dulbecco’s Modified Eagle Medium/Nutrient Mixture F-12 (DMEM/F-12; Gibco, Thermo Fisher Scientific, Waltham, MA, USA) supplemented with 10% foetal bovine serum (Capricorn Scientific, Ebsdorfergrund, Germany) and 1% antibiotic–antimycotic (Capricorn Scientific, Ebsdorfergrund, Germany). Cultures were maintained at 37 °C in a humidified atmosphere containing 5% CO_2_, and the medium was replaced every 2–3 days.

For post-thaw recovery, cells were transferred into complete growth medium and centrifuged at 250× *g* for 5 min. The cell pellet was resuspended and seeded into T25 culture flasks. Before experimental use, the cells were passaged once after recovery. At approximately 70–80% confluence, cells were washed with DPBS, detached using TrypLE Select Enzyme and collected by centrifugation at 300× *g* for 5 min. The cells were then resuspended in complete growth medium.

Cell concentration and viability were determined using a haemocytometer and 0.4% trypan blue exclusion before seeding. Viable cells were diluted to the seeding density required for each experimental activity.

### 4.6. VEGF Quantification in HUVEC Culture Supernatants

#### 4.6.1. Scaffold Preconditioning and HUVEC Seeding

Activity 1 evaluated soluble VEGF concentrations in HUVEC culture supernatants after 4 days of culture with F1, F2 and F3 scaffolds. The experimental conditions comprised HUVEC-seeded F1–F3 scaffolds, corresponding scaffold-only controls, a scaffold-free HUVEC cell-only group and a medium-only control.

Before cell seeding, the crosslinked scaffolds were equilibrated to room temperature, rinsed with sterile DPBS and exposed to ultraviolet light for surface decontamination. The scaffolds were subsequently immersed in complete EGM-2 medium and incubated at 37 °C for 1 h before seeding, consistent with previously reported scaffold preconditioning procedures [55].

The scaffold-only and medium-only controls were included to account for VEGF present in the supplemented culture medium and for formulation-specific scaffold-associated effects on soluble VEGF measurements.

#### 4.6.2. HUVEC Seeding and Supernatant Collection

HUVECs at passages 3–5 were resuspended in complete EGM-2 medium and seeded dropwise onto each scaffold in a 24-well plate at a density of 1.0 × 10^5^ cells per scaffold. This seeding density was selected within the range used for endothelial cell culture on 3D scaffolds [56].

The seeded scaffolds were incubated at 37 °C and 5% CO_2_ for 45 min to allow initial attachment. Complete EGM-2 medium was then added gently along the wall of each well to a final volume of 300 µL. The upper scaffold surface was not completely submerged. The cell-only group received the same number of HUVECs in 300 µL of medium without a scaffold. The scaffold-only and medium-only controls were maintained under the same culture conditions.

All groups were cultured for 4 days without medium replacement. On Day 4, culture supernatants were collected without washing the scaffolds or pipetting directly over their surfaces. Samples were transferred to microcentrifuge tubes and stored at −20 °C until analysis. All samples were analysed together in a single ELISA run to minimise inter-assay variation. Representative scaffold placement during Activity 1 is shown in Figure S12a.

#### 4.6.3. VEGF ELISA

VEGF concentrations were measured using a Human VEGF SimpleStep ELISA Kit (ab222510, Abcam, Cambridge, UK) according to the manufacturer’s instructions. All reagents were equilibrated to room temperature before use. VEGF standards were prepared by serial dilution using Sample Diluent NS containing the specified enhancer, and concentrations were determined using a four-parameter logistic (4PL) regression model. A representative standard curve is provided in Figure S1.

A preliminary dilution assessment was conducted to ensure that sample signals fell within the assay range. Supernatants from the HUVEC-seeded scaffold groups and corresponding scaffold-only controls were analysed without dilution. Supernatants from the HUVEC cell-only and medium-only groups were diluted fourfold before analysis. Interpolated concentrations from these diluted samples were multiplied by the dilution factor before further processing.

Standards and samples were added to the pre-coated wells with the one-step detection mixture and incubated for 1 h at room temperature on a microplate shaker at 150 rpm. The wells were washed three times, after which TMB substrate was added and incubated for 10 min at room temperature on a shaker at 150 rpm in the dark. The reaction was terminated using the supplied stop solution. Absorbance was measured at 450 nm using a Varioskan LUX multimode microplate reader (Thermo Fisher Scientific, Waltham, MA, USA) following a 1 min plate-shaking step.

#### 4.6.4. VEGF Quantification and Background Correction

Raw optical-density values were obtained using SkanIt software, version 7.1 (Thermo Fisher Scientific, Waltham, MA, USA) and corrected by subtracting the zero standard supplied with the ELISA kit. VEGF concentrations were interpolated from the 4PL standard curve.

For each scaffold-containing sample, the background-corrected VEGF concentration was calculated as:

(1) VEGF_corrected_ = VEGF_cell+scaffold_ − mean(VEGF_scaffold only_),

where mean(VEGF_scaffold only_) represents the mean concentration measured in the scaffold-only controls for the corresponding formulation.

For the scaffold-free reference condition:

(2) VEGF_HUVEC only_ = VEGF_cell only_ − mean(VEGF_medium only_).

The HUVEC-only group was reported as a scaffold-free 2D reference and was not included in the primary statistical comparison among F1, F2 and F3.

Each formulation comprised six independently cultured biological samples (*N* = 6). Each supernatant was measured once by ELISA, resulting in six measurements per formulation (*n* = 6), with no technical assay replicates. Under matched initial cell-seeding and culture conditions, the resulting VEGF values were reported as background-corrected concentrations in the culture supernatants collected on Day 4.

#### 4.6.5. Statistical Analysis

Background-corrected VEGF concentrations were analysed using IBM Statistical Package for the Social Sciences (SPSS) Statistics, version 30.0.0.0 (IBM Corp., Armonk, NY, USA). Normality was assessed using the Shapiro–Wilk test, and homogeneity of variance was evaluated using Levene’s test.

Because the assumptions of normality and equal variance were satisfied, differences among F1, F2 and F3 were analysed using one-way analysis of variance followed by Tukey’s HSD test. The HUVEC-only reference was presented descriptively and was not included in the one-way ANOVA. Data are presented as mean ± standard deviation, and *p* < 0.05 was considered statistically significant. Graphs were prepared using GraphPad Prism, version 10.6.0 (GraphPad Software, Boston, MA, USA).

### 4.7. Endothelial Induction and Immunofluorescence Analysis

#### 4.7.1. hPDLSC Seeding and Endothelial Induction

Before cell seeding, the scaffolds were equilibrated to room temperature, rinsed with sterile DPBS, exposed to ultraviolet light for surface decontamination and pre-wetted in complete hPDLSC growth medium at 37 °C for 1 h. Thin F1, F2 and F3 scaffolds measuring 10 × 10 × 1 mm were used to facilitate confocal imaging. hPDLSCs were seeded dropwise onto each scaffold at a density of 5 × 10^3^ cells/cm^2^ and incubated at 37 °C and 5% CO_2_ for 45 min to allow initial attachment.

Complete growth medium was then added gently along the well wall to a volume of 200 µL without fully submerging the upper scaffold surface. After a further 2 h, the medium volume was increased to 300 µL. This staged addition was used to minimise disturbance of newly attached cells. Representative scaffold placement during Activity 2 is shown in Figure S12b.

Endothelial induction was initiated using a stepwise transition from complete growth medium to complete EGM-2 medium to minimise medium-switch-associated stress [57]. The day on which EGM-2 was first introduced was defined as endothelial induction Day 0. On Day 0, EGM-2 was added to the existing growth medium to produce a growth medium: EGM-2 ratio of 3:1. On Day 1, the medium was replaced with a fresh 1:1 mixture. From Day 2 onwards, the cultures were maintained in complete EGM-2 medium, which has been used to support endothelial-associated responses in mesenchymal stem-cell models [58]. Cultures were maintained for 21 days from the first introduction of EGM-2, with medium replacement three times per week.

2D HUVEC positive controls were seeded at the same nominal surface density and maintained in complete EGM-2 medium for 7 days before staining. 2D and scaffold-based non-induced hPDLSC controls were maintained in complete growth medium without endothelial induction for the corresponding culture period.

#### 4.7.2. Immunofluorescence Controls

The following controls were included to support interpretation of staining specificity and background fluorescence:

1. HUVECs cultured under 2D conditions as a positive endothelial staining reference;
2. Non-induced hPDLSCs cultured under 2D conditions;
3. Non-induced hPDLSCs cultured on F1, F2 and F3 scaffolds; and
4. Induced hPDLSC-seeded scaffolds processed without primary antibodies.

The secondary antibody-only controls were incubated with the corresponding secondary antibodies and DAPI under the same staining and imaging conditions as the induced scaffold groups. All control groups were evaluated qualitatively and were not included in the primary quantitative comparison among F1, F2 and F3.

VEGF was assessed as an angiogenic-related marker rather than an endothelial-specific marker, because VEGF may also be expressed by non-endothelial cell populations. CD31 was included as the more endothelial-associated marker because of its established vascular adhesion functions and localisation at endothelial cell membranes and intercellular junctions [31,35]. The two markers were therefore interpreted together when assessing endothelial-associated responses in hPDLSCs.

#### 4.7.3. Immunofluorescence Staining

For immunofluorescence staining, 3D scaffolds were transferred to glass-bottom 24-well plates (Greiner Bio-One, Kremsmünster, Austria), whereas 2D cultures were maintained in glass-bottom plates. Samples were washed twice with DPBS and fixed with 4% paraformaldehyde (Fisher Scientific, Waltham, MA, USA) for 15 min at room temperature. After three DPBS washes, the cells were permeabilised with 0.01% Triton X-100 (Sigma-Aldrich, St. Louis, MO, USA) for 7 min and blocked with 10% normal goat serum (Solarbio Life Sciences, Beijing, China) for 90 min.

Samples were incubated overnight at 4 °C with rabbit anti-VEGF antibody (ab52917, Abcam, Cambridge, UK; 1:500) and mouse anti-CD31/PECAM-1 antibody (ab9498, Abcam, Cambridge, UK; 1:300). The primary antibodies were diluted in 1% bovine serum albumin (Bioworld, Dublin, OH, USA) prepared in DPBS. For secondary antibody-only controls, the primary antibodies were omitted and replaced with 1% bovine serum albumin in DPBS.

After washing, samples were incubated for 1 h at room temperature in the dark with goat anti-rabbit IgG conjugated to Alexa Fluor 488 (ab150077, Abcam, Cambridge, UK; 1:500) and goat anti-mouse IgG conjugated to Alexa Fluor 594 (ab150116, Abcam, Cambridge, UK; 1:500), diluted in 1% bovine serum albumin. Nuclei were counterstained with DAPI (E-IR-R103, Elabscience, Houston, TX, USA) for 5 min. Samples were subsequently rinsed with DPBS, maintained in DPBS and imaged immediately under light-protected conditions to minimise fluorescence fading.

#### 4.7.4. Confocal Image Acquisition

Immunofluorescence images were acquired using a Nikon Eclipse Ti inverted microscope equipped with a Nikon A1 confocal system (Nikon, Tokyo, Japan). Excitation was performed using 405 nm, 488 nm and 561 nm laser lines for DAPI, Alexa Fluor 488 and Alexa Fluor 594, respectively. Sequential channel acquisition was used to reduce spectral overlap and photobleaching during multichannel imaging [59].

3D scaffold samples were imaged using a 20× objective with the cell-seeded surface facing the objective. Z-stacks were acquired at 1 µm intervals across 41 optical sections, corresponding to an axial depth of approximately 40 µm. Each fluorescence channel was converted to a 2D maximum-intensity projection before visualisation and quantitative analysis [38]. Representative single-plane images were acquired from the 2D controls for qualitative comparison.

Laser power, pinhole size, scanning resolution, detector settings and acquisition order were kept constant within each experimental model and imaging set.

#### 4.7.5. Image Processing and Quantitative Signal Analysis

Quantitative image analysis was performed for the endothelial-induced F1, F2 and F3 scaffold groups using Fiji/ImageJ, version 2.16.0. The 2D HUVEC positive control, 2D and 3D non-induced hPDLSC controls and secondary antibody-only controls were assessed qualitatively and were not included in the primary quantitative comparison.

For each scaffold, three fields of view were selected using a predefined random-field sampling approach. The scaffold surface was conceptually divided into sampling zones, and one field was acquired from each zone without reference to VEGF or CD31 signal intensity. Each ROI corresponded to the complete field captured using the 20× objective rather than a post-acquisition cropped region. Fields containing edge artefacts, physical defects or marked uneven illumination were excluded before analysis. The same acquisition settings were maintained for all 3D samples within each fluorescence channel.

Native ND2 files were imported into Fiji using the Bio-Formats plugin, with the original image metadata and spatial calibration retained. The DAPI, VEGF and CD31 channels were separated and processed independently. Each 41-section z-stack was converted to a 2D maximum-intensity projection. A median filter was applied using a radius of 1 pixel for DAPI and 2 pixels for VEGF and CD31.

Fixed channel-specific threshold settings were established before batch analysis and applied uniformly to all images within the corresponding channel. After thresholding, images were converted to binary format. The Fill Holes and Watershed functions were applied to restore enclosed signal regions and separate adjacent signal objects, respectively.

Particles were enumerated using the Analyse Particles function. The upper particle-size limit was set to infinity, and circularity was set to 0.00–1.00. Fixed minimum particle-size thresholds of 60 µm^2^ for DAPI, 50 µm^2^ for VEGF and 50 µm^2^ for CD31 were applied to all images. The number of detected DAPI-, VEGF- and CD31-associated signal objects was recorded for each field. The image-processing workflow is illustrated in Figure S13.

#### 4.7.6. Selection and Robustness Assessment of Particle-Size Thresholds

Minimum particle-size thresholds were selected before final batch analysis to reduce the inclusion of small noise-related objects while retaining visually identifiable biological signals. For each fluorescence channel, one representative image was randomly selected independently from each formulation dataset, giving three images per channel. The images selected for the DAPI, VEGF and CD31 assessments were not required to originate from the same scaffold samples.

Each selected image was reanalysed using adjacent candidate minimum-size thresholds while all other processing parameters were held constant, including the median filter, intensity threshold, binary processing functions, upper size limit and circularity range. DAPI-associated signals were evaluated at minimum thresholds of 50, 60 and 70 µm^2^. VEGF- and CD31-associated signals were evaluated at 40, 50, 60 and 70 µm^2^.

Percentage changes in particle counts between consecutive thresholds were calculated to assess the sensitivity of the output to modest parameter changes. The results indicated that increasing the threshold beyond the selected values produced larger reductions in particle counts in some representative images, particularly at 70 µm^2^. The final thresholds of 60 µm^2^ for DAPI and 50 µm^2^ for VEGF and CD31 were therefore selected as a balance between noise exclusion and signal retention. Detailed sensitivity analysis results are presented in Tables S20–S22.

#### 4.7.7. Signal Proportion Calculation

For each field, the VEGF- and CD31-associated particle counts were separately normalised to the DAPI-detectable nuclear particle count obtained from the corresponding image:

Marker-associated signal proportion (%) = [marker-associated signal count/corresponding DAPI-detectable nuclear signal count] × 100.(3)

The calculation was performed independently for VEGF and CD31 in each field. The three image-level proportions obtained from the same scaffold were then averaged to generate one scaffold-level biological replicate value. Each formulation therefore comprised three biological replicate values for statistical analysis (*N* = 3), derived from nine image-level measurements (*n* = 9).

The resulting proportions were interpreted as image-based measures of angiogenic-related or endothelial-associated signals relative to the detectable nuclear signal within the same field.

#### 4.7.8. Statistical Analysis

Statistical analysis was performed using IBM SPSS Statistics, version 30.0.0.0. Normality was assessed separately for each formulation using the Shapiro–Wilk test, and homogeneity of variance was evaluated using Levene’s test based on the mean. No significant violations of normality or homogeneity of variance were detected.

Differences in VEGF- and CD31-associated signal proportions among F1, F2 and F3 were analysed using one-way analysis of variance followed by Tukey’s HSD test for pairwise comparisons. Statistical significance was set at *p* < 0.05. Graphs were prepared using GraphPad Prism, version 10.6.0. Detailed assumption testing, ANOVA and post hoc results are presented in Tables S5–S8.

### 4.8. Osteogenic Induction and ALP Activity Assay

#### 4.8.1. Experimental Groups and Replicate Structure

Activity 3 was performed to compare early osteogenic-associated responses among the scaffold groups by measuring alkaline phosphatase (ALP) activity in lysates obtained from hPDLSC-seeded constructs over time. Three scaffold formulations with increasing HA content, namely F1, F2 and F3, were evaluated under osteogenic induction conditions. A 100% nano-hydroxyapatite block measuring 6 mm in diameter and 3 mm in height was included as a mineral-only material reference.

Corresponding non-induced scaffold controls were prepared by culturing hPDLSCs on F1, F2 and F3 scaffolds in complete growth medium without osteogenic supplements. 2D hPDLSC cultures maintained in osteogenic differentiation medium or complete growth medium were included as system-validation controls. These 2D controls were used to confirm osteogenic responsiveness under conventional monolayer culture conditions and were not treated as direct comparators for the 3D scaffold groups.

ALP activity was assessed on induction Days 1, 4, 7 and 10. At each time point, three independent biological samples were prepared for each group. Each lysate was analysed in duplicate wells, giving three biological replicate values per group and time point (*N* = 3). Duplicate assay readings from the same biological sample were averaged before statistical analysis.

#### 4.8.2. hPDLSC Preparation and Scaffold Preparation

hPDLSCs were maintained in complete growth medium at 37 °C in a humidified incubator with 5% CO_2_. Cells were passaged once after thawing and used during the exponential growth phase. Before seeding, cells were detached using TrypLE Select Enzyme, collected by centrifugation and resuspended in complete growth medium. Cell number and viability were determined using trypan blue exclusion.

For A3, printed 3D-HA–APO constructs were prepared using the smallest available preset scaffold template of the BIO X bioprinter, producing scaffolds measuring approximately 10 × 10 × 3 mm. Each construct was aseptically divided into four approximately equal segments, and one-quarter segments were used for the ALP assay. This was performed to better approximate the volume of the 100% HA reference block while retaining the same printed scaffold formulation.

Before cell seeding, the scaffold segments and HA blocks were equilibrated to room temperature, rinsed with sterile DPBS, exposed to ultraviolet light for surface decontamination and pre-wetted in complete hPDLSC growth medium.

#### 4.8.3. Cell Seeding in 2D and 3D Cultures

For 2D cultures, hPDLSCs were seeded in 24-well plates at 5 × 10^4^ cells/well in 400 µL of complete growth medium.

For 3D cultures, each scaffold segment or HA block was placed individually in a 48-well plate. A 10 µL cell suspension containing 1 × 10^5^ hPDLSCs was carefully pipetted onto the upper surface of each construct. The constructs were incubated for 45 min at 37 °C and 5% CO_2_ to allow initial cell attachment. Subsequently, 200 µL of complete growth medium was gently added along the well wall without directly disturbing the seeded surface. After a further 2 h of incubation, the final medium volume was adjusted to 300 µL.

#### 4.8.4. Osteogenic Induction and Time-Point Definition

Osteogenic induction was initiated by replacing complete growth medium with osteogenic differentiation medium. The first medium replacement with osteogenic differentiation medium was defined as induction Day 0.

For 2D cultures, induction was initiated when cells reached approximately 70–80% confluence. The cultures were washed twice with DPBS, and 400 µL of osteogenic differentiation medium was added per well.

For 3D constructs, osteogenic induction was initiated 24 h after seeding to allow initial cell attachment and recovery. Growth medium was carefully aspirated and replaced with 300 µL of osteogenic differentiation medium. Non-induced scaffold controls received complete growth medium at the corresponding time point. Samples were collected on induction Days 1, 4, 7 and 10.

#### 4.8.5. Culture Maintenance and Sample Collection

Media were refreshed three times per week according to group allocation. Medium aspiration and replacement were performed gently to minimise cell loss from 3D constructs and to avoid disrupting the cell layer in 2D cultures.

Samples were collected destructively on induction Days 1, 4, 7 and 10. Separate sample sets were prepared for each time point, and each sample was harvested only once. At each collection time point, culture medium was removed, and samples were washed twice with cold DPBS. 2D cultures were processed directly in the wells, whereas scaffold segments and HA blocks were processed as intact constructs. Lysates were either analysed on the same day or stored at −80 °C until ALP activity analysis. Stored lysates were thawed once before analysis.

#### 4.8.6. Lysate Preparation

For 3D scaffold segments and HA block samples, cell–construct composites were processed as intact constructs to recover intracellular ALP from cells associated with the 3D matrices. Each construct was mechanically disrupted in 250 µL of ALP assay buffer on ice using a glass Dounce homogeniser. Where necessary, the original culture well and homogenisation apparatus were rinsed with additional assay buffer, and the washings were combined with the primary lysate.

The lysates were centrifuged at 12,000× *g* for 15 min at 4 °C to remove insoluble scaffold residues and cellular debris. Only the clarified supernatants were used for ALP activity measurement to minimise optical interference during the colourimetric assay. Representative lysate clarification during ALP sample preparation is shown in Figure S14.

For 2D cultures, cells were lysed directly in the wells by adding 80 µL of ALP assay buffer after DPBS washing. The cells were detached and disrupted using a sterile cell scraper, and the lysates were transferred to microcentrifuge tubes. The lysates were centrifuged at 4 °C for 15 min, and the supernatants were collected for ALP measurement.

#### 4.8.7. ALP Activity Assay

ALP activity was quantified using a colourimetric ALP assay kit (ab83369, Abcam, Cambridge, UK) according to the manufacturer’s instructions, with minor modifications. The p-nitrophenyl phosphate (pNPP) working solution was freshly prepared before use by dissolving one pNPP tablet in 2.7 mL of ALP assay buffer and was protected from light.

Clarified lysates were loaded into 96-well plates and adjusted with ALP assay buffer to a final sample loading volume of 80 µL per well. For 2D samples, 30 µL of clarified lysate was loaded per assay well and adjusted to 80 µL with assay buffer. For 3D scaffold and HA block samples, 80 µL of clarified lysate was loaded directly unless dilution was required. Samples were analysed in duplicate wells.

The enzymatic reaction was initiated by adding 50 µL of pNPP working solution to each sample and standard well. Plates were incubated at 25 °C for 60 min protected from light. The reaction was then terminated by adding 20 µL of stop solution, and absorbance was measured at 405 nm using the Varioskan LUX multimode microplate reader. Raw optical density (OD) values were acquired using SkanIt software, version 7.1.

Standards were freshly prepared for each assay run, and one standard curve was generated for each plate. The zero standard was used as the reagent blank. Its mean absorbance was subtracted from all standards and samples before interpolation from the standard curve. Representative ALP standard curves are provided in Figure S8.

#### 4.8.8. Background Correction for Coloured Scaffold Lysates

Most clarified lysates were colourless after centrifugation and were corrected only by subtraction of the reagent blank. However, some 3D non-induced scaffold lysates showed faint residual colouration, likely due to retention of phenol red-containing growth medium within the scaffold matrix. For these samples, matched sample-specific background controls were included where applicable.

Background control wells contained the same lysate volume and dilution factor as the corresponding sample wells. Stop solution was added before pNPP addition to prevent enzymatic colour development while preserving sample-associated optical background. The corrected absorbance for coloured non-induced scaffold lysates was calculated as:

OD_corrected,NI_ = OD_sample,blank-corrected_ − OD_background,blank-corrected_. (4)

This correction was applied only to samples with visible matrix-associated colour interference.

#### 4.8.9. ALP Activity Calculation

The amount of p-nitrophenol (pNP) generated in each reaction well was determined by interpolation from the corresponding blank-corrected standard curve. ALP activity was expressed as milliunits per millilitre (mU/mL) and calculated as:

(5) ALP activity (mU/mL) = [*B*/(*T* × *V*)] × *D* × 1000,

where *B* is the amount of p-nitrophenol (pNP) generated in the reaction well (nmol), *T* is the reaction time (min), *V* is the original sample volume loaded into the reaction well (µL), and *D* is the dilution factor. The factor of 1000 converts the sample volume from microlitres to millilitres. The resulting ALP activity values were expressed per millilitre of clarified lysate loaded into the assay.

#### 4.8.10. Statistical Analysis

For the primary A3 analysis, ALP activity in the induced F1, F2, F3 and 100% HA reference groups was analysed. Duplicate technical readings from the same biological sample were averaged to generate one biological replicate value before statistical analysis. Each group at each time point therefore comprised three biological replicate values.

ALP activity was first analysed using two-way ANOVA to assess the effects of day, scaffold group and their interaction. Because a significant Day × Scaffold Group interaction was detected, between-group differences were further examined within each time point. Normality was assessed using the Shapiro–Wilk test, and homogeneity of variance was evaluated using Levene’s test based on the mean. Day 1, Day 4 and Day 7 data were analysed using one-way ANOVA followed by Tukey’s HSD test. Day 10 data were analysed using Welch ANOVA followed by Games–Howell post hoc comparisons because homogeneity of variance was not satisfied. Statistical significance was set at *p* < 0.05.

The 2D induced, 2D non-induced and 3D non-induced groups were used as supportive controls to confirm induction responsiveness and baseline ALP activity under the corresponding culture conditions. These controls were presented descriptively and were not included in the primary formulation comparison.

Statistical analysis was performed using IBM SPSS Statistics, version 30.0.0.0. Graphs were prepared using GraphPad Prism, version 10.6.0. Detailed descriptive statistics, assumption testing, omnibus tests and post hoc comparisons for the primary ALP analysis are provided in Tables S9–S16. Supportive control data are provided in Figure S9 and Tables S17 and S18.

## 5. Conclusions

This study demonstrated formulation-dependent differences in selected in vitro angiogenic-related and early osteogenic outcomes of 3D-HA–APO scaffolds under matched initial seeding and culture conditions. Among the three formulations evaluated, F3 showed the most favourable overall biological response profile across the selected angiogenic-related and early osteogenic outcomes. It was associated with the highest background-corrected soluble VEGF concentration, the strongest VEGF- and CD31-associated signal profile, and generally the highest ALP activity at the later osteogenic induction time points.

These findings support prioritising F3 for further investigation within the current scaffold platform. They also provide a basis for formulation-level optimisation of the 3D-HA–APO system. Future studies integrating physicochemical characterisation, endpoint cellular assessment and functional angiogenic and later osteogenic evaluation will help determine which formulation-associated properties underlie the observed response profile and whether these early advantages are maintained in more complex models relevant to periodontal and alveolar bone regeneration.

## Data Availability

The data supporting the findings of this study are included in this article and its Supplementary Materials. Additional data are available from the corresponding author upon reasonable request. Detailed compositional information for the 3D-HA–APO bioink is not publicly disclosed due to intellectual property and confidentiality considerations.

## Supplementary Materials

The following supporting information can be downloaded at: https://www.mdpi.com/article/10.3390/ijms27177531/s1.

## Abbreviations

The following abbreviations are used in this manuscript:

2D	Two-dimensional
3D	Three-dimensional
3D-HA–APO	Three-dimensional hydroxyapatite–acrylated palm olein
4PL	Four-parameter logistic
ALP	Alkaline phosphatase
ANOVA	Analysis of variance
APO	Acrylated palm olein
CD31	Cluster of differentiation 31
DAPI	4′,6-Diamidino-2-phenylindole
DMEM	Dulbecco’s Modified Eagle Medium
DPBS	Dulbecco’s phosphate-buffered saline
EBM-2	Endothelial basal medium-2
EGM-2	Endothelial growth medium-2
ELISA	Enzyme-linked immunosorbent assay
HA	Hydroxyapatite
hPDLSCs	Human periodontal ligament stem cells
HSD	Honestly significant difference
HUVECs	Human umbilical vein endothelial cells
mU	Milliunit
ns	Not significant
OD	Optical density
PECAM-1	Platelet endothelial cell adhesion molecule-1
pNP	p-nitrophenol
pNPP	p-nitrophenyl phosphate
ROI	Region of interest
SD	Standard deviation
SPSS	Statistical Package for the Social Sciences
UKM	Universiti Kebangsaan Malaysia
*v*/*v*	Volume/volume
VEGF	Vascular endothelial growth factor
*w*/*v*	Weight/volume
