# Supplementary material for: Preliminary Formulation-Dependent Angiogenesis-Related and Early Osteogenic Responses to Three-Dimensional Bioprinted Hydroxyapatite–Acrylated Palm Olein Scaffolds: An In Vitro Study
Source: Int J Mol Sci. 2026 Aug 22;27(17):7531. doi: 10.3390/ijms27177531 (PMC13566144; doi:10.3390/ijms27177531)
Supplement: Supplementary file 1 [file ijms-27-07531-s001.zip › ijms-4462001-supplementary.pdf]

Supplementary Materials

for

Preliminary Formulation-Dependent Angiogenic-Related and Early Osteogenic Responses to Three-Dimensional Bioprinted Hydroxyapatite–Acrylated Palm Olein Scaffolds: An In Vitro Study

Xi Chen, Nik Madihah Nik Azis, Syafira Masri and Masfueh Razali

S1. Background Data for VEGF Quantification

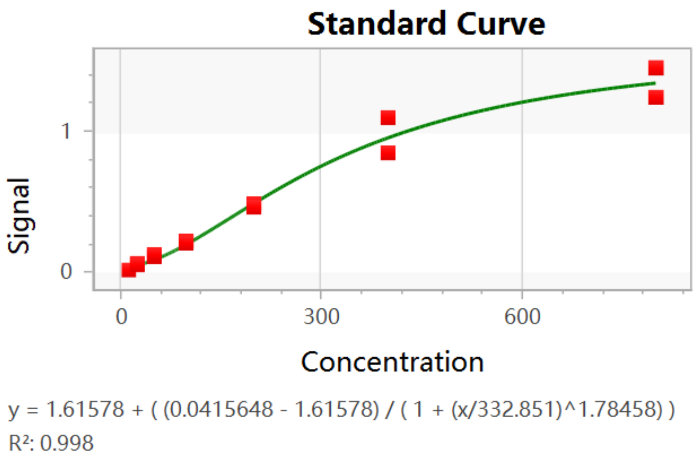

Figure S1. Representative standard curve used for vascular endothelial growth factor (VEGF) quantification by enzyme-linked immunosorbent assay (ELISA). VEGF concentrations were calculated using a four-parameter logistic regression model. The coefficient of determination was $R^2 = 0.998$.

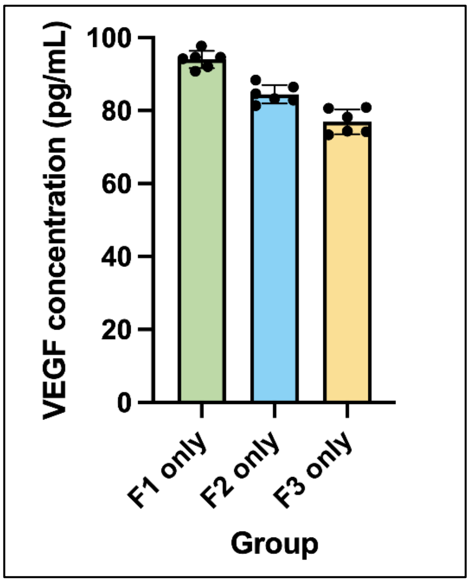

Figure S2. Vascular endothelial growth factor (VEGF) concentrations detected in the scaffold-only control groups after 4 days of incubation. F1 only, F2 only and F3 only represent cell-free three-dimensional hydroxyapatite–acrylated palm olein (3D-HA–APO) scaffolds containing 5%, 7% and 10% (w/v) hydroxyapatite (HA), respectively. The mean value of each scaffold-only control group was used for background correction of the corresponding human umbilical vein endothelial cell (HUVEC)-seeded scaffold measurements. Data are presented as mean ± standard deviation (SD).

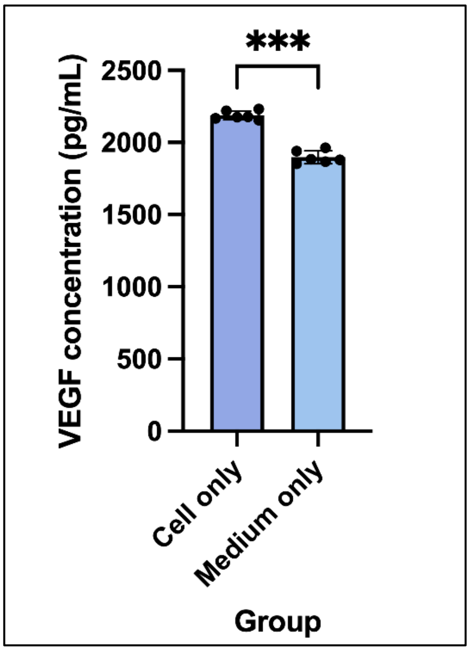

Figure S3. Raw vascular endothelial growth factor (VEGF) concentrations detected in the human umbilical vein endothelial cell (HUVEC) cell-only and medium-only control groups. The mean VEGF concentration of the medium-only controls was subtracted from each HUVEC cell-only value to obtain the background-corrected HUVEC-only reference presented in the main text. Data are presented as

mean ± standard deviation (SD) from six independently cultured samples per condition ($N = 6$). The cell-only and medium-only groups were compared using an independent-samples t-test, $t(10) = 13.220$, $p < 0.001$, Hedges' $g = 7.043$ (95 % CI, 3.817–10.238). The mean difference was 290.08 pg/mL. ***$p < 0.001$.

Table S1. Descriptive statistics for background-corrected vascular endothelial growth factor (VEGF) concentrations in the scaffold-containing groups and the human umbilical vein endothelial cell (HUVEC)-only reference.

Group	n	Mean ± SD (pg/mL)	95% CI	Minimum–maximum
F1 (5% HA)	6	77.68 ± 15.83	61.07–94.29	57.37–102.92
F2 (7% HA)	6	203.16 ± 11.62	190.97–215.36	189.04–218.22
F3 (10% HA)	6	270.86 ± 28.25	241.21–300.51	240.61–318.62
HUVEC-only reference	6	290.08 ± 30.57	257.99–322.16	257.15–333.49

Note: Values are background-corrected as described in the Materials and Methods. The HUVEC-only group was presented descriptively and was not included in the one-way analysis of variance (ANOVA) comparing F1–F3. HA, hydroxyapatite; SD, standard deviation; CI, confidence interval; n, number of raw measurements.

Table S2. Normality assessment and one-way analysis of variance (ANOVA) for background-corrected vascular endothelial growth factor (VEGF) concentrations.

Group	Shapiro–Wilk W	*p* value
F1	0.985	0.974
F2	0.943	0.680
F3	0.907	0.416

Source	Sum of squares	df	Mean square	*F*	*p* value	Eta squared (η^2)
Between groups	115294.451	2	57647.226	146.094	<0.001	0.951
Within groups	5918.852	15	394.590			
Total	121213.304	17				

Note: Eta squared was calculated as the between-group sum of squares divided by the total sum of squares.

Test	Statistic	df1	df2	*p* value
Levene's test based on the mean	1.074	2	15	0.367

Table S3. Tukey's honestly significant difference (HSD) pairwise comparisons of background-corrected vascular endothelial growth factor (VEGF) concentrations among the scaffold-containing groups.

Comparison	Mean difference (pg/mL)	95% CI	Adjusted p value
F2 − F1	125.48	95.69–155.27	<0.001
F3 − F1	193.18	163.39–222.97	<0.001
F3 − F2	67.70	37.91–97.49	<0.001

S2. Supplementary Statistical Results for Immunofluorescence Quantification

S2.1. Descriptive Statistics Based on Biological Replicate Means

Quantitative immunofluorescence analysis was based on three biological replicates per scaffold formulation. Three regions of interest were analysed from each scaffold and averaged to generate one biological replicate value. Descriptive statistics for 4′,6-diamidino-2-phenylindole (DAPI)-detectable nuclear signals and vascular endothelial growth factor (VEGF)- and cluster of differentiation 31 (CD31)-associated signal proportions are presented in **Table S4**.

Table S4. Descriptive statistics for 4′,6-diamidino-2-phenylindole (DAPI)-detectable nuclear signals and vascular endothelial growth factor (VEGF)- and CD31-associated signal proportions based on biological replicate means.

Scaffold formulation	DAPI-detectable nuclear signals	VEGF-associated signals (%)	CD31-associated signals (%)
F1 (5% HA)	105.33 ± 9.21	44.41 ± 5.48	26.17 ± 1.17
F2 (7% HA)	77.78 ± 18.26	60.23 ± 7.90	35.54 ± 4.56
F3 (10% HA)	78.00 ± 15.62	74.88 ± 8.35	56.84 ± 3.50

Note: Data are presented as mean ± standard deviation (SD); *N* = 3 biological replicates per formulation. Each biological replicate value was calculated as the mean of three regions of interest from one scaffold. HA, hydroxyapatite; CD31, cluster of differentiation 31.

S2.2. Assumption Testing

Normality was assessed separately for each formulation using the Shapiro–Wilk test, and homogeneity of variance was assessed using Levene's test based on the mean. The results are presented in **Table S5**.

Table S5. Assumption testing for vascular endothelial growth factor (VEGF)- and cluster of differentiation 31 (CD31)-associated signal proportions.

Outcome	Group	Shapiro–Wilk W	*p*	Levene statistic	*p*
VEGF-associated signals (%)	F1 (5% HA)	0.960	0.613	0.693	0.536
F2 (7% HA)	0.773	0.052			
F3 (10% HA)	0.853	0.250			

CD31-associated signals (%)	F1 (5% HA)	0.900	0.384	1.629	0.272
	F2 (7% HA)	0.988	0.788		
	F3 (10% HA)	0.963	0.630		

Note: Shapiro–Wilk tests were performed separately for each formulation. Levene's test was performed across the three formulations for each outcome, with df1 = 2 and df2 = 6. No significant departures from normality or homogeneity of variance were detected at $p < 0.05$.

S2.3. One-Way ANOVA for VEGF- and CD31-Associated Signals

One-way ANOVA was used to assess formulation-dependent differences in VEGF- and CD31-associated signal proportions. The results are summarised in **Table S6**.

Table S6. One-way analysis of variance (ANOVA) results for vascular endothelial growth factor (VEGF)- and CD31-associated signal proportions.

Outcome	Between-group df	Within-group df	*F*	*p*	Eta squared (η^2)
VEGF-associated signals (%)	2	6	12.885	0.007	0.811
CD31-associated signals (%)	2	6	64.541	<0.001	0.956

Note: Eta squared was calculated as the between-group sum of squares divided by the total sum of squares.

S2.4. Tukey HSD Pairwise Comparisons

Tukey's HSD test was used for post hoc pairwise comparisons following significant omnibus ANOVA results. The pairwise comparisons are presented in **Table S7** and **Table S8**.

Table S7. Tukey honestly significant difference (HSD) comparisons for vascular endothelial growth factor (VEGF)-associated signal proportions.

Comparison	Mean difference (%)	95% CI	*p*
F2 vs. F1	15.82	−2.60 to 34.24	0.086
F3 vs. F1	30.47	12.05 to 48.89	0.005
F3 vs. F2	14.65	−3.77 to 33.07	0.110

Table S8. Tukey honestly significant difference (HSD) comparisons for CD31-associated signal proportions.

Comparison	Mean difference (%)	95% CI	*p*
F2 vs. F1	9.37	0.88 to 17.86	0.034
F3 vs. F1	30.68	22.19 to 39.17	<0.001
F3 vs. F2	21.31	12.82 to 29.80	<0.001

S3. Supplementary Immunofluorescence Images for Activity 2

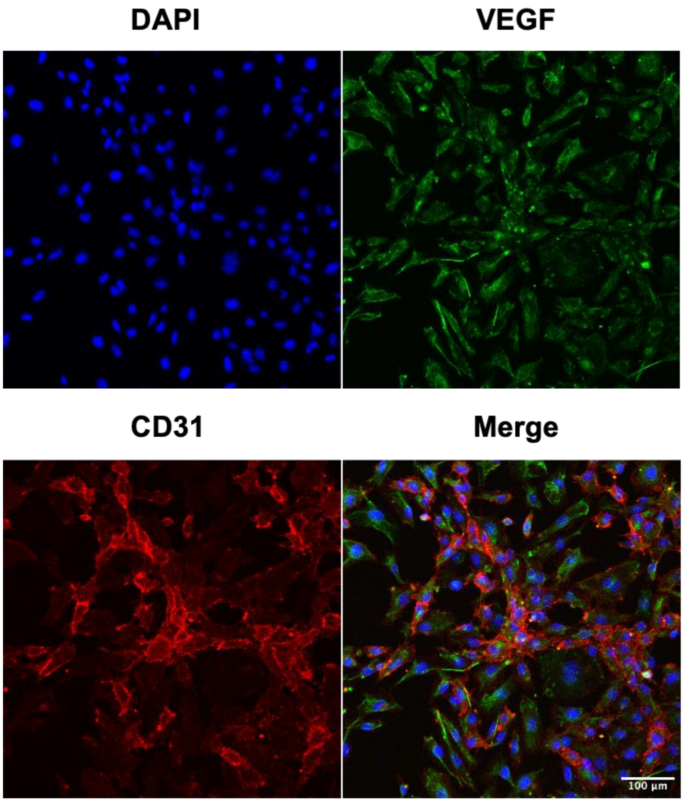

Figure S4. Human umbilical vein endothelial cell (HUVEC) positive control staining for vascular endothelial growth factor (VEGF) and CD31. Representative confocal immunofluorescence images of HUVECs cultured for 7 days and stained for 4′,6-diamidino-2-phenylindole (DAPI), VEGF and CD31. Scale bar = 100 μm. CD31, cluster of differentiation 31.

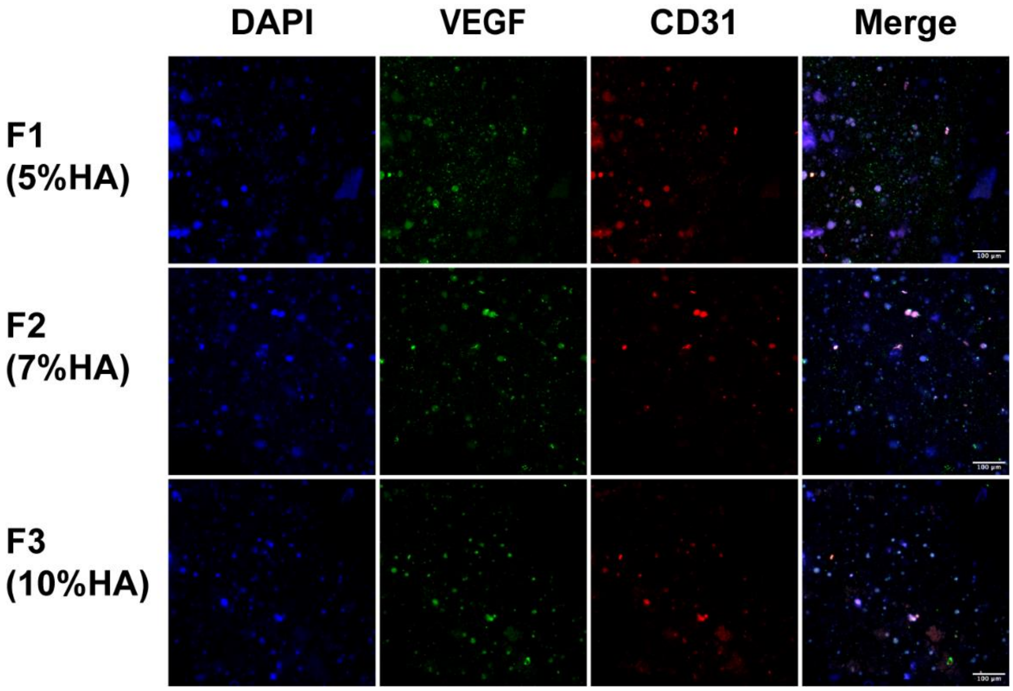

Figure S5. Additional representative immunofluorescence images of endothelial-induced human periodontal ligament stem cells (hPDLSCs) cultured on three-dimensional hydroxyapatite–acrylated palm olein (3D-HA–APO) scaffolds. Additional confocal maximum-intensity projection images are shown for F1, F2 and F3 after 21 days of endothelial induction. F1, F2 and F3 contained 5%, 7% and 10% (w/v) hydroxyapatite (HA), respectively. Cells were stained for 4′,6-diamidino-2-phenylindole (DAPI), vascular endothelial growth factor (VEGF) and CD31. Scale bar = 100 μm. CD31, cluster of differentiation 31.

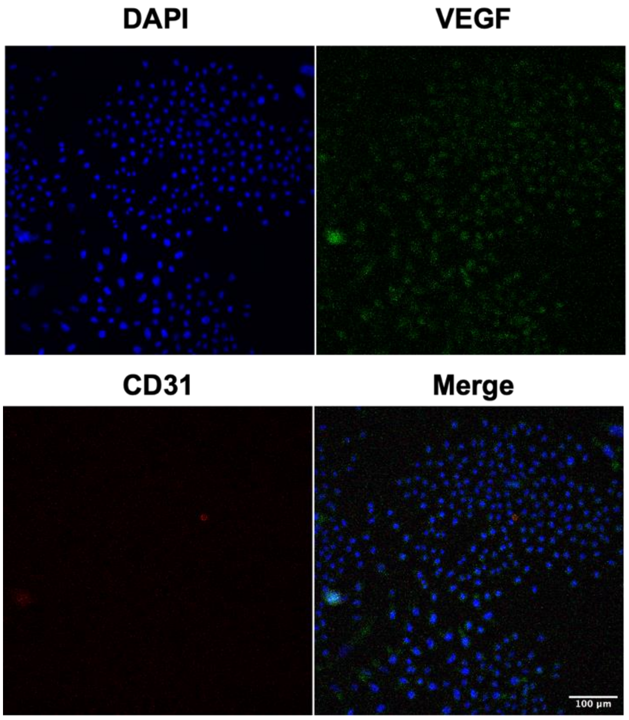

Figure S6. Representative confocal immunofluorescence images of non-induced human periodontal ligament stem cells (hPDLSCs). Cells were stained for vascular endothelial growth factor (VEGF; green), CD31 (red) and 4′,6-diamidino-2-phenylindole (DAPI; blue) after 21 days of culture in complete medium. CD31, cluster of differentiation 31.

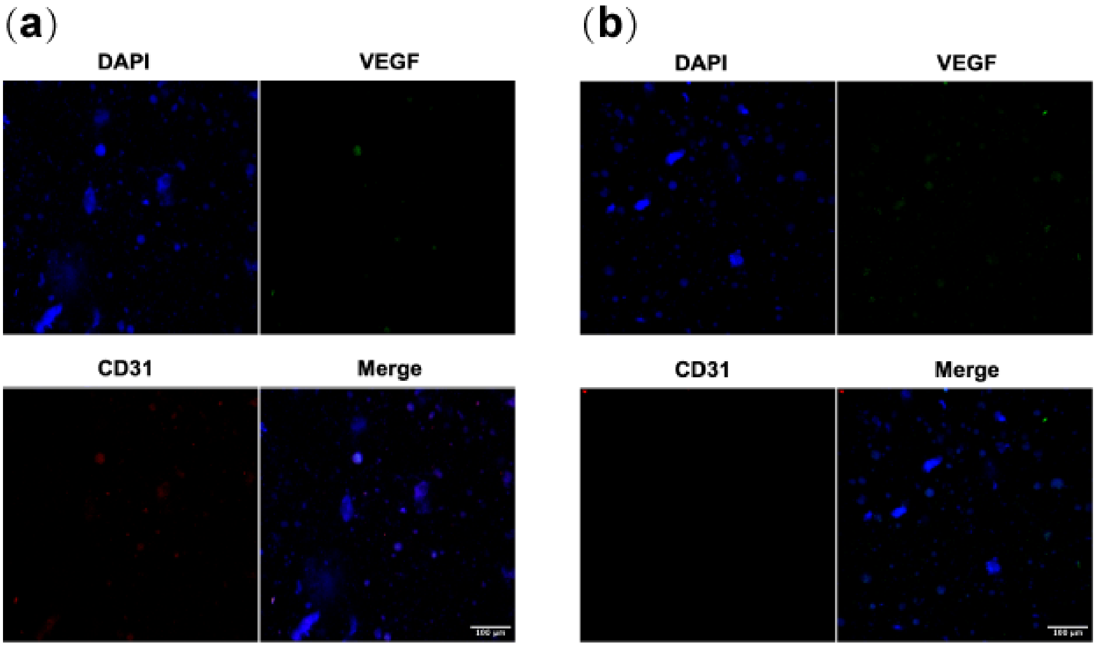

Figure S7. Representative immunofluorescence images of three-dimensional (3D) non-induced and secondary antibody-only controls. (**a**) Secondary antibody-only control on an induced F2 scaffold, prepared by omitting the primary antibodies. (**b**) Non-induced human periodontal ligament stem cells (hPDLSCs) cultured on an F2 three-dimensional hydroxyapatite–acrylated palm olein (3D-HA–APO)

scaffold for 21 days in complete growth medium. Cells were stained or imaged for 4′,6-diamidino-2-phenylindole (DAPI), vascular endothelial growth factor (VEGF) and CD31 as indicated. Scale bar = 100 μm. CD31, cluster of differentiation 31.

S4. Supplementary Results for ALP Activity

S4.1. Alkaline Phosphatase (ALP) Standard Curves

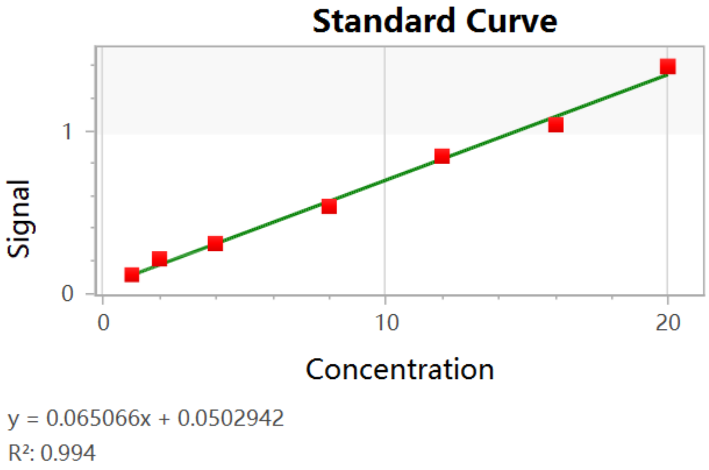

Figure S8. Representative linear standard curves used for alkaline phosphatase (ALP) activity determination. Standard curves were generated using the Alkaline Phosphatase Assay Kit, and absorbance signal was plotted against standard concentration. Linear regression equations and R^2 values are shown within each panel.

S4.2. Descriptive Statistics for ALP Activity

Descriptive statistics for alkaline phosphatase (ALP) activity in the induced F1, F2, F3 and 100% hydroxyapatite (HA) reference groups are summarised in **Table S9**.

Table S9. Descriptive statistics for alkaline phosphatase (ALP) activity in induced scaffold and hydroxyapatite (HA) reference groups.

Time Point	Group	Mean	SD
Day 1	F1	0.390	0.011
	F2	0.397	0.008
	F3	0.401	0.011
	HA	0.359	0.008
Day 4	F1	1.695	0.052
	F2	1.543	0.017
	F3	1.988	0.060
	HA	1.794	0.027
Day 7	F1	4.739	0.099
	F2	5.315	0.117
	F3	5.828	0.110

Day 10	HA	5.065	0.183
	F1	5.613	0.049
	F2	6.937	0.451
	F3	9.737	0.453
	HA	5.444	0.201

Note: Data are presented as mean ± standard deviation (SD). Each group at each time point comprised three biological replicates.

S4.3. Two-Way Analysis of Variance (ANOVA) for ALP Activity

Two-way ANOVA was used to evaluate the effects of day, scaffold group and their interaction on ALP activity. The results are summarised in **Table S10**.

Table S10. Two-way analysis of variance (ANOVA) results for alkaline phosphatase (ALP) activity.

Source	df	*F*	*p*	Partial η^2
Day	3	3331.181	<0.001	0.997
Scaffold Group	3	147.679	<0.001	0.933
Day × Scaffold Group	9	77.505	<0.001	0.956

Note: The analysis included F1, F2, F3 and the 100% hydroxyapatite (HA) reference construct across Days 1, 4, 7 and 10. Error df = 32.

S4.4. Time-Point-Specific Assumption Testing

Normality was assessed using the Shapiro–Wilk test for each group at each time point. Homogeneity of variance was assessed using Levene's test based on the mean. The results are presented in **Table S11**.

Table S11. Assumption testing for time-point-specific alkaline phosphatase (ALP) comparisons.

Time Point	Group	Shapiro–Wilk W	p	Levene Statistic	*p*	Test Selected
Day 1	F1	0.773	0.052	0.451	0.724	One-way ANOVA
	F2	0.948	0.560			
	F3	0.949	0.566			
	HA	0.870	0.297			
Day 4	F1	1.000	0.996	1.011	0.437	One-way ANOVA
	F2	0.925	0.472			
	F3	0.995	0.870			
	HA	0.914	0.432			
Day 7	F1	0.935	0.508	0.827	0.515	One-way ANOVA
	F2	0.994	0.856			
	F3	0.999	0.942			
	HA	0.898	0.380			
Day 10	F1	0.995	0.863	4.666	0.036	Welch ANOVA
	F2	0.876	0.314			
	F3	0.852	0.246			
	HA	0.920	0.452			

Note: Shapiro–Wilk tests were performed separately for each group. Levene's test was performed across F1, F2, F3 and hydroxyapatite (HA) within each time point using the test based on the mean. For Levene's test, df1 = 3 and df2 = 8. Day 10 was analysed using Welch analysis of variance (ANOVA) because homogeneity of variance was not satisfied.

S4.5. Time-Point-Specific Omnibus Tests

Because a significant Day × Scaffold Group interaction was detected, between-group differences were examined separately within each time point. The time-point-specific omnibus tests are summarised in **Table S12**.

Table S12. Time-point-specific omnibus tests for alkaline phosphatase (ALP) activity.

Time Point	Test	df1	df2	Statistic	*p*
Day 1	One-way ANOVA	3	8	12.435	0.002
Day 4	One-way ANOVA	3	8	57.685	<0.001
Day 7	One-way ANOVA	3	8	36.741	<0.001
Day 10	Welch ANOVA	3	3.501	65.844	0.001

Note: Day 1, Day 4 and Day 7 met the assumptions for one-way ANOVA. Day 10 was analysed using Welch analysis of variance (ANOVA) because Levene's test indicated unequal variances.

S4.6. Post Hoc Pairwise Comparisons

Tukey's honestly significant difference (HSD) test was used for Day 1, Day 4 and Day 7. Games–Howell post hoc comparisons were used for Day 10. Pairwise comparisons are presented in **Table S13–Table S16**.

Table S13. Tukey honestly significant difference (HSD) pairwise comparisons for alkaline phosphatase (ALP) activity on Day 1.

Comparison	Mean Difference	95% CI	*p*
F2 vs. F1	0.007	−0.017 to 0.032	0.774
F3 vs. F1	0.011	−0.014 to 0.035	0.535
HA vs. F1	−0.031	−0.056 to −0.007	0.015
F3 vs. F2	0.003	−0.021 to 0.028	0.971
HA vs. F2	−0.038	−0.063 to −0.014	0.004
HA vs. F3	−0.042	−0.066 to −0.017	0.003

Table S14. Tukey honestly significant difference (HSD) pairwise comparisons for alkaline phosphatase (ALP) activity on Day 4.

Comparison	Mean Difference	95% CI	*p*
F2 vs. F1	−0.152	−0.263 to −0.040	0.010
F3 vs. F1	0.293	0.182 to 0.405	<0.001
HA vs. F1	0.099	−0.012 to 0.211	0.081
F3 vs. F2	0.445	0.334 to 0.556	<0.001

HA vs. F2	0.251	0.140 to 0.363	<0.001
HA vs. F3	−0.194	−0.305 to −0.083	0.002

Table S15. Tukey honestly significant difference (HSD) pairwise comparisons for alkaline phosphatase (ALP) activity on Day 7.

Comparison	Mean Difference	95% CI	*p*
F2 vs. F1	0.576	0.233 to 0.919	0.003
F3 vs. F1	1.089	0.746 to 1.432	<0.001
HA vs. F1	0.326	−0.017 to 0.670	0.062
F3 vs. F2	0.513	0.170 to 0.856	0.006
HA vs. F2	−0.250	−0.593 to 0.093	0.170
HA vs. F3	−0.763	−1.106 to −0.420	<0.001

Table S16. Games–Howell pairwise comparisons for alkaline phosphatase (ALP) activity on Day 10.

Comparison	Mean Difference	95% CI	*p*
F2 vs. F1	1.323	−0.447 to 3.094	0.088
F3 vs. F1	4.124	2.346 to 5.902	0.009
HA vs. F1	−0.170	−0.904 to 0.564	0.584
F3 vs. F2	2.801	1.297 to 4.304	0.006
HA vs. F2	−1.493	−2.956 to −0.030	0.048
HA vs. F3	−4.293	−5.764 to −2.823	0.003

Table S17. Descriptive statistics for quantifiable alkaline phosphatase (ALP) activity in 2D-induced human periodontal ligament stem cells (hPDLSCs).

Time point	N	Mean ± SD ALP activity (mU/mL)	Minimum	Maximum
Day 4	3	1.68 ± 0.06	1.62	1.74
Day 7	3	5.57 ± 0.07	5.49	5.63
Day 10	3	5.81 ± 0.17	5.65	5.98

Note: Some 2D ALP readings were below the lower quantifiable range of the standard curve and were therefore not included in inferential statistical analysis. Data are presented for quantifiable 2D-induced samples from Day 4 onwards. SD, standard deviation; mU/mL, milliunits per millilitre.

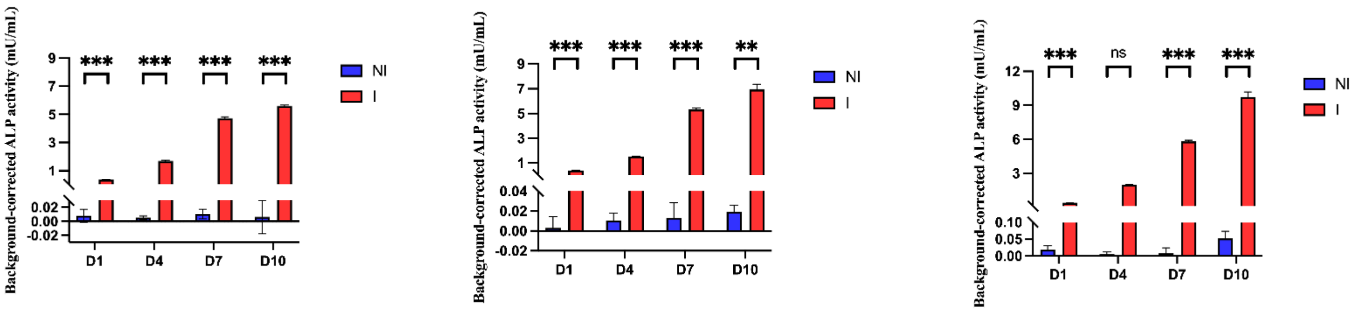

(**a**) (**b**) (**c**)

Figure S9. Supportive comparisons of alkaline phosphatase (ALP) activity between osteogenic-induced and corresponding non-induced three-dimensional hydroxyapatite–acrylated palm olein scaffold groups: (**a**) F1 [5% (w/v) HA]; (**b**) F2 [7% (w/v) HA]; and (**c**) F3 [10% (w/v) HA]. Data are presented as mean ± standard deviation (SD) based on biological replicate values ($N = 3$ per condition at each time point). Comparisons were performed using independent-samples t-tests, Welch *t*-tests where homogeneity of variance was not satisfied, or an exact Mann–Whitney *U* test where parametric assumptions were not met. Induced and non-induced groups differed at all evaluated time points except F3 on Day 4 ($U = 0.000$, exact $p = 0.100$). Exact test statistics and two-sided *p* values are provided in Table S18. NI, non-induced; I, osteogenic-induced; ns, not significant; **$p < 0.01$; ***$p < 0.001$.

Table S18. Comparisons of alkaline phosphatase (ALP) activity between osteogenic-induced and corresponding non-induced scaffold groups.

Formulation	Day	Statistical test	Statistic	df	Mean difference (mU/mL)	Two-sided *p* value
F1	1	Independent-samples t-test	$t = 47.369$	4	0.382	<0.001
F1	4	Independent-samples t-test	$t = 56.567$	4	1.690	<0.001
F1	7	Welch t-test	$t = 82.660$	2.018	4.728	<0.001
F1	10	Independent-samples t-test	$t = 178.830$	4	5.607	<0.001
F2	1	Independent-samples t-test	$t = 49.636$	4	0.394	<0.001
F2	4	Independent-samples t-test	$t = 144.865$	4	1.533	<0.001
F2	7	Independent-samples t-test	$t = 78.023$	4	5.302	<0.001
F2	10	Welch t-test	$t = 26.534$	2.001	6.917	0.001
F3	1	Independent-samples t-test	$t = 39.782$	4	0.382	<0.001
F3	4	Exact Mann–Whitney *U* test	$U = 0.000$	—	—	0.100

F3	7	Independent-samples t-test	t = 91.182	4	5.819	<0.001
F3	10	Welch t-test	t = 36.958	2.009	9.685	<0.001

Note: Positive mean differences indicate higher ALP activity in the osteogenic-induced group than in the corresponding non-induced group. Welch *t*-tests were used where homogeneity of variance was not satisfied. The exact two-sided p value is reported for the Mann–Whitney U test. Each condition comprised three biological replicates (N = 3).

Table S19. Within-group temporal comparisons of alkaline phosphatase (ALP) activity.

Group	Comparison	Mean difference (later − earlier), mU/mL	95 % CI	*p* value
F1	Day 4 vs Day 1	1.305	1.145 to 1.465	<0.001
F1	Day 7 vs Day 1	4.349	4.189 to 4.509	<0.001
F1	Day 10 vs Day 1	5.223	5.064 to 5.383	<0.001
F1	Day 7 vs Day 4	3.044	2.884 to 3.204	<0.001
F1	Day 10 vs Day 4	3.919	3.759 to 4.079	<0.001
F1	Day 10 vs Day 7	0.875	0.715 to 1.035	<0.001
F2	Day 4 vs Day 1	1.146	1.092 to 1.200	<0.001
F2	Day 7 vs Day 1	4.918	4.454 to 5.381	<0.001
F2	Day 10 vs Day 1	6.539	4.734 to 8.344	0.004
F2	Day 7 vs Day 4	3.772	3.321 to 4.223	<0.001
F2	Day 10 vs Day 4	5.394	3.592 to 7.195	0.006
F2	Day 10 vs Day 7	1.622	−0.016 to 3.259	0.051
F3	Day 4 vs Day 1	1.587	1.361 to 1.814	<0.001
F3	Day 7 vs Day 1	5.427	4.997 to 5.858	<0.001
F3	Day 10 vs Day 1	9.336	7.525 to 11.148	0.002
F3	Day 7 vs Day 4	3.840	3.499 to 4.180	<0.001
F3	Day 10 vs Day 4	7.749	5.987 to 9.511	0.002
F3	Day 10 vs Day 7	3.909	2.248 to 5.571	0.008
HA	Day 4 vs Day 1	1.435	1.340 to 1.531	<0.001
HA	Day 7 vs Day 1	4.706	3.976 to 5.436	0.001
HA	Day 10 vs Day 1	5.085	4.285 to 5.886	0.001
HA	Day 7 vs Day 4	3.271	2.564 to 3.978	0.002
HA	Day 10 vs Day 4	3.650	2.871 to 4.429	0.002
HA	Day 10 vs Day 7	0.379	−0.262 to 1.020	0.217

Note: Positive mean differences indicate higher ALP activity at the later time point. F1 comparisons were performed using Tukey's honestly significant difference test. F2, F3 and HA comparisons were performed using Games–Howell tests because homogeneity of variance was not satisfied. Each group comprised three independent biological replicates at each time point (N = 3). CI, confidence interval; HA, hydroxyapatite.

S5. Supplementary Methods

S5.1. Replicate Structure and Measurement Hierarchy

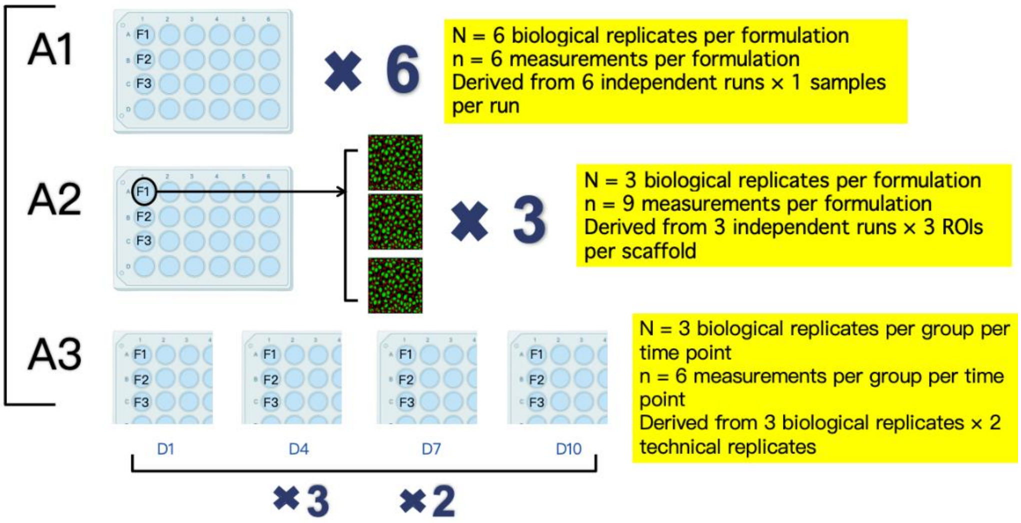

Figure S10. Schematic summary of the replicate structure and measurement hierarchy used in Activities 1–3. For Activity 1, each formulation comprised six independently cultured scaffold-associated biological samples, and each supernatant was measured once by enzyme-linked immunosorbent assay (ELISA). For Activity 2, each formulation comprised three biological replicates, with three regions of interest (ROIs) analysed per scaffold and averaged to generate one biological replicate value. For Activity 3, each group at each time point comprised three biological replicates, and each lysate was measured in duplicate assay wells. Additional control and reference groups are not shown for clarity.

S5.2. Scaffold Fabrication Workflow

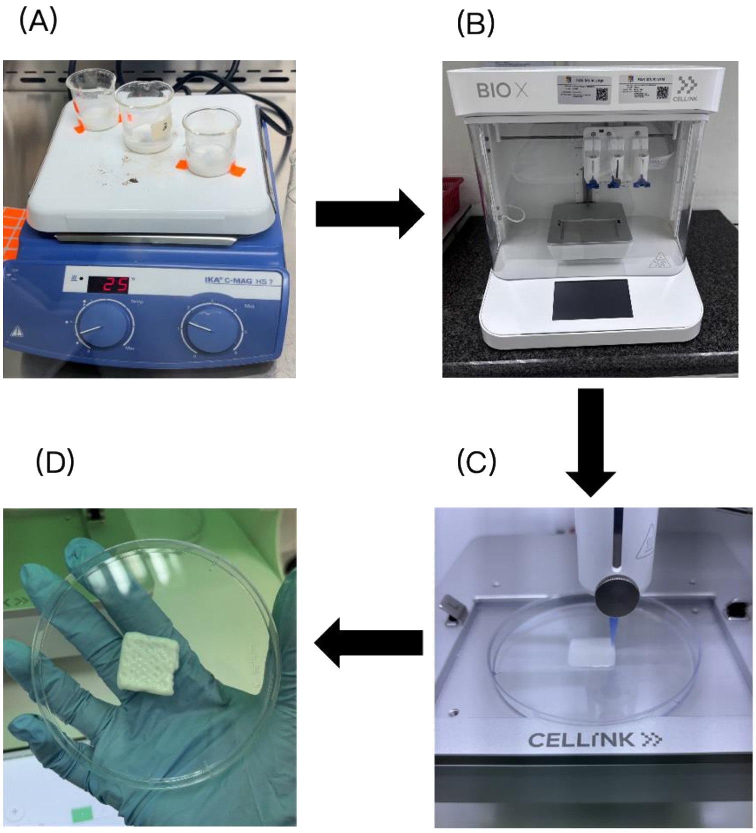

Figure S11. Representative workflow for fabrication of the three-dimensional hydroxyapatite–acrylated palm olein (3D-HA–APO) scaffolds. (**A**) Homogenisation of the hydroxyapatite–acrylated palm olein (HA–APO) composite ink under magnetic stirring. (**B**) BIOX extrusion-based three-dimensional (3D) bioprinter used for scaffold fabrication. (**C**) Layer-by-layer extrusion of the composite ink onto a sterile substrate. (**D**) Representative 3D-HA–APO construct immediately after printing.

S5.3. Scaffold Placement During Biological Assays

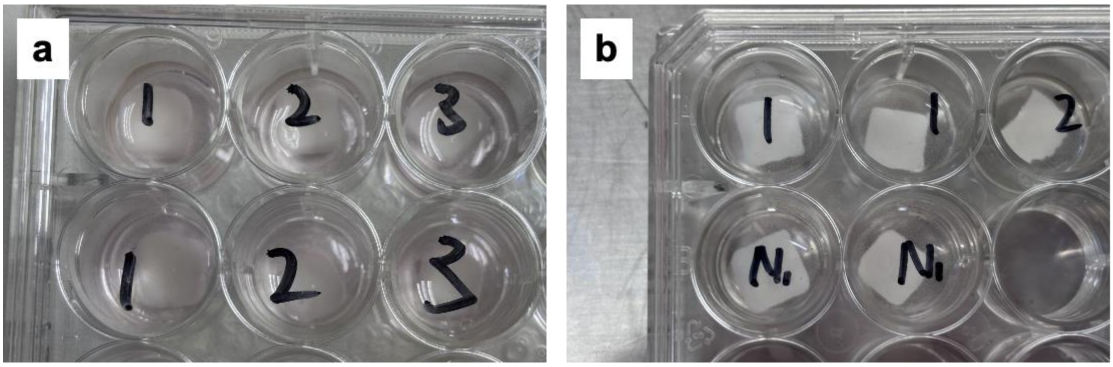

Figure S12. Representative wet three-dimensional hydroxyapatite–acrylated palm olein (3D-HA–APO) scaffold constructs used during biological assay setup. (**a**) Scaffold constructs placed individually in a 24-well culture plate for human umbilical vein endothelial cell (HUVEC) seeding and supernatant collection in Activity 1. (**b**) Scaffold constructs maintained in a 24-well culture plate during Activity 2 before transfer to glass-bottom culture plates for immunofluorescence imaging.

S5.4. Selection and Robustness Assessment of Minimum Particle-Size Thresholds

Candidate minimum particle-size thresholds were evaluated to determine whether modest changes in the lower size limit materially altered particle counts. Representative images from F1, F2 and F3 were reanalysed using a series of adjacent minimum-size thresholds. Percentage changes in particle counts between consecutive thresholds were calculated to assess parameter sensitivity. The final thresholds were selected to reduce the inclusion of small noise-related particles while retaining visually identifiable biological signals. The representative images selected for the 4′,6-diamidino-2-phenylindole (DAPI), vascular endothelial growth factor (VEGF) and cluster of differentiation 31 (CD31) assessments were drawn independently and were not restricted to the same scaffold samples. The sensitivity analyses for the DAPI, VEGF and CD31 channels are summarised in **Table S20–Table S22**.

Table S20. Sensitivity analysis of the minimum particle-size threshold for the 4′,6-diamidino-2-phenylindole (DAPI) channel.

Formulation	50 μm²	60 μm²	70 μm²	Change, 50→60	Change, 60→70
F1	98	91	80	−7.1%	−12.1%
F2	79	74	68	−6.3%	−8.1%
F3	71	61	57	−14.1%	−6.6%

Table S21. Sensitivity analysis of the minimum particle-size threshold for the vascular endothelial growth factor (VEGF) channel.

Formulation	40 μm²	50 μm²	60 μm²	70 μm²	Change, 40→50	Change, 50→60	Change, 60→70
F1	57	47	42	31	−17.5%	−10.6%	−26.2%
F2	45	40	38	26	−11.1%	−5.0%	−31.6%
F3	74	66	63	55	−10.8%	−4.5%	−12.7%

Table S22. Sensitivity analysis of the minimum particle-size threshold for the cluster of differentiation 31 (CD31) channel.

Formulation	40 μm²	50 μm²	60 μm²	70 μm²	Change, 40→50	Change, 50→60	Change, 60→70
F1	22	20	17	10	−9.1%	−15.0%	−41.2%
F2	40	36	33	23	−10.0%	−8.3%	−30.3%
F3	55	48	45	38	−12.7%	−6.2%	−15.6%

S5.5. Fiji Image-Processing Workflow

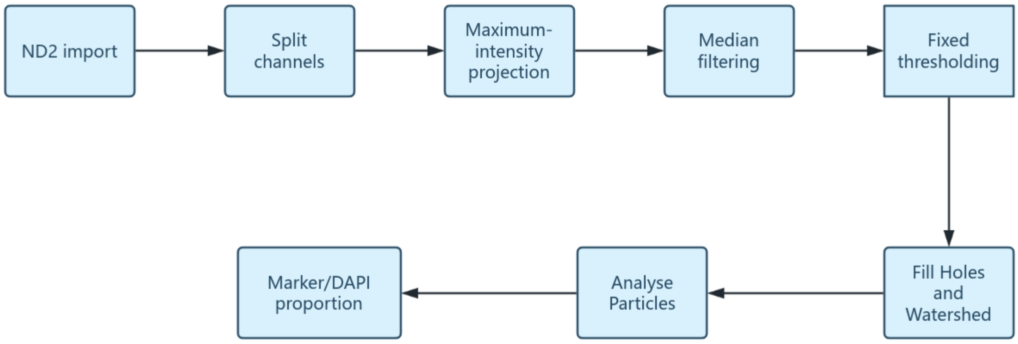

Figure S13. Schematic workflow for immunofluorescence image processing in Fiji and subsequent marker-associated signal proportion calculation. Native Nikon NIS-Elements Document (ND2) files were imported using Bio-Formats, separated into individual fluorescence channels and converted to maximum-intensity projections. Channel-specific median filtering, fixed thresholding, binary processing and particle analysis were performed in Fiji. The resulting marker-associated signal counts were then normalised to the corresponding 4′,6-diamidino-2-phenylindole (DAPI)-detectable nuclear signal counts to calculate image-level signal proportions.

S5.6. Representative Lysate Clarification During Alkaline Phosphatase (ALP) Sample Preparation

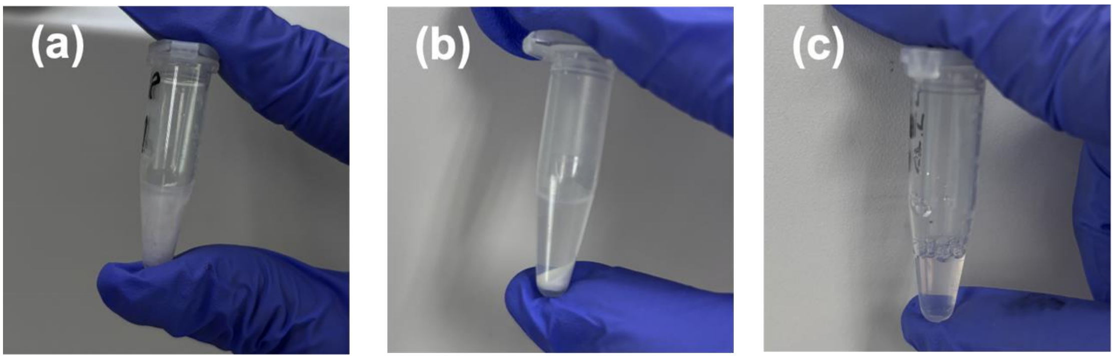

Figure S14. Representative lysate clarification during alkaline phosphatase (ALP) sample preparation. (**a**) Turbid homogenised scaffold lysate after mechanical disruption in ALP assay buffer; (**b**) phase separation after centrifugation at 12,000 × g for 15 min at 4 °C, with scaffold debris sedimented at the bottom; (**c**) clarified supernatant collected for ALP activity measurement.
